# Recent Advances in Strategies for the Cloning of Natural Product Biosynthetic Gene Clusters

**DOI:** 10.3389/fbioe.2021.692797

**Published:** 2021-07-13

**Authors:** Wenfang Wang, Guosong Zheng, Yinhua Lu

**Affiliations:** ^1^College of Life Sciences, Shanghai Normal University, Shanghai, China; ^2^Shanghai Engineering Research Center of Plant Germplasm Resources, College of Life Sciences, Shanghai Normal University, Shanghai, China

**Keywords:** natural product, biosynthetic gene cluster (BGC), high capacity vector, DNA assembly, direct cloning

## Abstract

Microbial natural products (NPs) are a major source of pharmacological agents. Most NPs are synthesized from specific biosynthetic gene clusters (BGCs). With the rapid increase of sequenced microbial genomes, large numbers of NP BGCs have been discovered, regarded as a treasure trove of novel bioactive compounds. However, many NP BGCs are silent in native hosts under laboratory conditions. In order to explore their therapeutic potential, a main route is to activate these silent NP BGCs in heterologous hosts. To this end, the first step is to accurately and efficiently capture these BGCs. In the past decades, a large number of effective technologies for cloning NP BGCs have been established, which has greatly promoted drug discovery research. Herein, we describe recent advances in strategies for BGC cloning, with a focus on the preparation of high-molecular-weight DNA fragment, selection and optimization of vectors used for carrying large-size DNA, and methods for assembling targeted DNA fragment and appropriate vector. The future direction into novel, universal, and high-efficiency methods for cloning NP BGCs is also prospected.

## Introduction

Natural products (NPs) produced by microbes are a major source of pharmacological agents and industrially useful compounds. With the spread of drug-resistant pathogens rendering widely used antibiotics ineffective, the discovery of new NPs has become an urgent necessity (Atanasov et al., 2021). The development of next-generation sequencing technology has led to the genomes of a vast array of culturable microorganisms being sequenced in recent years. Through analysis of sequenced microbial genomes, a remarkably large number of orphan biosynthetic gene clusters (BGCs) have been discovered, which represent a treasure trove of novel bioactive compounds with potential pharmacological relevance (Kang and Kim, 2021). However, translating these putative BGCs into specialized compounds is a challenge, as the majority of NP BGCs are either poorly or not at all expressed in native hosts under defined conditions (Li et al., 2021). Further, it has been estimated that over 99% of environmental microbes are recalcitrant to culture under laboratory conditions (Daniel, 2005). Now, metagenomics has emerged as a strategic approach to explore uncultivated microbes from environment (Daniel, 2005), which also revealed the presence of a vast array of NP BGCs. In addition, to facilitate the exploration of NP sources from uncultured microbes, many innovative techniques for targeted or high-throughput cultivation of novel microorganisms are emerging rapidly. Nevertheless, further development of cultivation technologies is still required (Lewis et al., 2021).

In the past decades, efforts have been committed to explore this treasure trove and a number of efficient strategies for activating silent gene clusters have been developed, among which the heterologous expression of NP BGCs has been most widely used (Kang and Kim, 2021). An advantage of this strategy is that once a novel metabolite appears in the surrogate host cell wherein the BGC has been introduced, it can be ascribed to the gene cluster with a high degree of confidence (Hu et al., 2016). A prerequisite for heterologous expression is to clone the target BGC into a suitable vector. Traditional library construction method is sequence-independent and has been proven to be efficient for cloning NP BGCs. Recently, it has been successfully employed for cloning NP BGCs larger than 150 kb using the bacterial artificial chromosome (BAC) (Zhang L. et al., 2017; Hashimoto et al., 2018; Sun et al., 2018). However, it requires considerable screening, which is time-consuming and laborious. In order to directly clone the target BGCs, researchers have developed a variety of DNA cloning or assembly methods, including *in vitro* DNA assembly (restriction enzyme-mediated assembly, recombination-based assembly, enzyme-independent DNA assembly), as well as *in vivo* direct cloning methods, such as Red/ET-mediated recombination in *Escherichia coli* and transformation-associated recombination (TAR) cloning in *Saccharomyces cerevisiae* (Abbasi et al., 2020; Lin et al., 2020).

**FIGURE 1:**
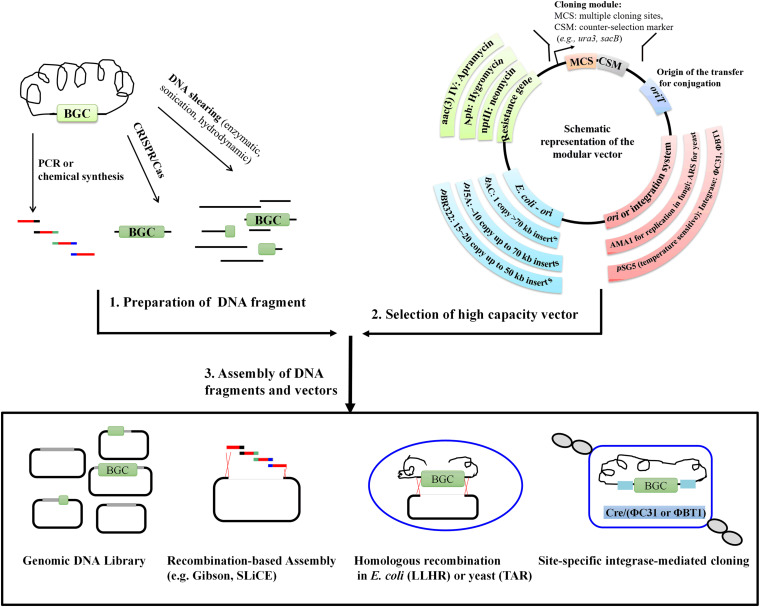
The general workflow for the cloning of natural product BGCs.

The previously reported *in vitro*, *in vivo*, or even *vitro*/*vivo* hybrid technologies for cloning large DNA fragments have distinct mechanisms, advantages, as well as drawbacks. However, regardless of the methods employed, it is necessary to prepare high-quality and high-molecular-weight DNA as well as to select suitable vectors. Further, efficient strategies for assembling large DNA fragments and vectors are required (Figure 1). The cloned BGCs usually have to be refactored in order to become more compatible with the heterologous host. In this review, we will focus on recent developments of the process for high-molecular-weight DNA fragment preparation, vectors used for carrying large-size DNA and methods for assembling target BGCs and vectors, and have a prospect on novel, universal and high-efficiency cloning methods for large-size DNA.

## Preparation of High Quality and High-Molecular-Weight DNA

Microbial NP BGCs normally range from 10 to 150 kb in length. Thus, methods for preparing high-quality and high-molecular-weight DNA are critical for successful cloning of intact BGCs. Fundamentally, genomic DNA extraction from microorganisms mainly involves three steps: (1) lysis of the cell wall or membrane using chemical disruption [e.g., SDS (sodium dodecyl sulfate)], enzymatic lysis (e.g., lysozyme and proteinase K), or physical disruption (e.g., manually grinding, sonication); (2) removal of all other unwanted cell components including cell wall debris, proteins, polysaccharides, and other metabolic substances by CTAB (cetyltrimethylammonium bromide) and/or phenol-chloroform; (3) recovery of the pure genomic DNA by ethanol precipitation, spin column-based technique, or magnetic bead-based strategy (Park, 2007; Varma et al., 2007). Integrity of genomic DNA may be mainly affected by the mechanical shearing and endogenous DNases. In these methods, proteinase K, phenol, and EDTA could suppress DNase activity, to a certain extent, enhancing the integrity of genomic DNA (Varma et al., 2007). To prevent mechanical shearing of DNA, microbial cells (e.g., protoplasts) can be embedded in low-melting-point agarose gels in the form of plugs, resulting in the preparation of megabase-sized DNA (Zhang M. et al., 2012). However, this process takes 3 days, and the operation is complex. A novel method for genomic DNA extraction, which involves cell grinding in liquid nitrogen, lysis with SDS-base buffer, and purification using carboxylated magnetic beads, was recently developed. Using this method, up to 80 kb DNA fragments could be prepared rapidly (∼1 h) and efficiently (Mayjonade et al., 2016). By fine-tuning three critical parameters, including the grinding duration and vibrational frequency, as well as lysis temperature and duration, the sizes of genomic DNA fragments ranging from 79 to 145 kb can be obtained (Penouilh-Suzette et al., 2020). More detailed information of the methods for genome DNA extraction can refer to a recent review (Gomez-Acata et al., 2019). However, no method can be universally applicable to all microorganisms. In general, researchers need to modify or blend different methods to obtain DNA of desired quality (Varma et al., 2007).

In the case where the genome sequence information of native hosts is under-characterized (e.g., environmental DNA), target BGCs can only be obtained through the construction of a genomic DNA library and subsequent screening via PCR or the identification of corresponding products via heterologous expression. Currently, three methods are available for DNA fragmentation for the construction of large-sized fragment libraries, including enzymatic digestion, sonication, and hydrodynamic shearing (Ignatov et al., 2019). Among them, sonication and hydrodynamic methods randomly disrupt the genome, which may cause the shearing of intact BGCs into different segments (Ignatov et al., 2019). So, enzymatic digestion is the most widely used for DNA fragmentation of library construction.

Currently, more and more microbial genome sequences are being published. For cloning small- to mid-sized BGCs, long-amplicon PCR could be used to amplify BGCs fragments, and then entire BGCs were obtained by DNA assembly (Greunke et al., 2018). However, as the length of NP BGCs increases, the probability of mutations introduced by PCR also increases. Fortunately, the development of genome editing tool CRISPR-Cas (clustered regularly interspaced short palindromic repeat-CRISPR-associated protein) system has made it possible to isolate the exact sequence of target BGCs (Lee et al., 2015; Jiang and Zhu, 2016). With the aid of Cas9 endonuclease, DNA segment of desired sizes can be obtained through generating the double strand breaks (DSBs) at specific sites within the genome guided by sgRNA. For example, bacterial cultures (e.g., *Escherichia coli*) was embedded in a low-melting-point agarose gel plug, treated with lysozyme and proteinase K, and subsequently washed to remove cellular components, leaving behind genomic DNA. Finally, the plug was transferred into cleavage buffer containing Cas9 and corresponding sgRNA pairs, which were designed to target genome segments of different lengths (50, 75, 100, 150, 200 kb). Clear DNA bands at the expected lengths were observed using pulsed-field gel electrophoresis (PFGE) assessment (Jiang et al., 2015). Recently, CISMR (CRISPR-mediated isolation of specific megabase-sized regions of the genome), which enables the targeted isolation of contiguous megabase-sized segments of the mouse genome, has also been developed by improving *in vitro* CRISPR specificity with the aid of both Target Finder and ZiFIT Targeter software to design 17 base sgRNA other than traditional 20 base target sequences (Bennett-Baker and Mueller, 2017). Further, a highly sensitive novel method for the simultaneous separation and concentration of high-molecular-weight DNA fragments was established by optimizing the formulation of viscoelastic liquids and engineering a capillary system. It was successfully used to isolate a 31.5 kb DNA fragment from the complicated 450 Mb *Medicago truncatula* genome with the aid of Cas9 cleavage (Milon et al., 2019).

The quality of DNA fragments can be analyzed via fragment analyzer or horizontal agarose gel electrophoresis. DNA fragments of desired sizes can be separated and extracted through multiple rounds of PFGE with different ramped pulse times (Clos and Zander-Dinse, 2019). Regardless of the cloning method used, sufficient amounts of DNA fragments are indispensable. Therefore, the preparation of high-quality and high-molecular-weight DNA fragments is recognized as a critical step in gene cluster cloning (Sapojnikova et al., 2017).

## Vectors for BGC Cloning

Given that most NP BGCs are of relatively large in length, appropriate vector systems capable of carrying the entire gene cluster as well as shuffling these genetic segments between different hosts are necessary. Since the first generation of general cloning vectors was introduced in 1973, a variety of high-capacity vectors have been developed so far (Bajpai, 2014). Despite the dazzling choice of commercial and other available vectors, cloning vector selection can be determined by several key criteria, such as the BGC size and GC content, vector copy number, host compatibility of different vectors, selection markers, and multiple cloning sites. Several types of high-capacity vectors are available for cloning large DNA fragments, including cosmid and artificial chromosomes, such as the fungal artificial chromosome (FAC), yeast artificial chromosome (YAC), bacterial artificial chromosome (BAC), and P1 phage artificial chromosome (PAC) (Monaco and Larin, 1994; Bajpai, 2014; Bok et al., 2015; Clevenger et al., 2017).

### Cosmid

Cosmid vectors, the first generation of high-capacity vectors used in genome research, are hybrids of plasmid and phage λ vector. As such, the cosmid vector encodes *cos* sequences required for packaging large fragments into the λ capsid and propagates their DNA as a virus or plasmid in the host cell. Since the possibility for cloning large fragments in cosmid vectors was first confirmed in 1979, they have been widely used for the construction of genomic libraries of various biological species, including Drosophila, mice, and humans. The construction of cosmid library is relatively simple and has been widely applied for cloning various NP BGCs. However, as the cosmid library requires tedious screening, it is necessary to combine high-throughput screening and sequencing methods. For example, the anisomycin BGC from *Streptomyces hygrospinosus* was identified using a bioactivity-guided high-throughput method for cosmid library screening (Zheng et al., 2017). Recently, CONKAT-seq (co-occurrence network analysis of targeted sequences) was used to uncover the potential of the rare BGCs from millions of cosmid clones harboring metagenomic DNA inserts (Libis et al., 2019).

Cosmid vectors can accommodate up to approximately 40 kb of DNA. They are multicopy plasmids in *E. coli* that facilitate DNA isolation and *in vitro* manipulation. However, loss of inserted DNA sometimes occurs within cosmid clones, which may be indicative of sequences that are instability in *E. coli* or of the transcription/translation products of the sequences are toxic to *E. coli*, particularly at a high copy number.

An F factor cosmid (fosmid), which contains a replicon derived from F factor and exists at a low/single copy number in *E. coli*, is more stable than its conventional cosmid counterpart (Kim et al., 1992). In addition, fosmid has an inducible *oriV* replication start point for high copy propagation, if necessary. Recently, a fosmid library containing 10,656 clones of metagenomic DNA isolated from the ATII (the Red Sea brine pool, Atlantis II Deep) lower convective layer (LCL) was functionally screened, and the products of two putative NP BGCs were detected to exhibit antibacterial and anticancer effects (Ziko et al., 2019). Typically, a cosmid or fosmid vector can only accept relatively small BGCs (up to 45 kb), which greatly hampers their application in cloning large NP BGCs.

### Artificial Chromosome

To address the limitation of cosmids, artificial chromosomal vectors, including YAC, PAC, BAC, and FAC, which harbor the carrying capacity of 100∼350 kb, have been used for cloning NP BGCs.

#### Yeast Artificial Chromosome

YAC vectors contain two copies of yeast telomeres for chromosomal stability, a yeast centromere for segregation, a yeast ARS (autonomously replicating sequence) for replication, and appropriate markers for the selection of recombinant molecules (Burke et al., 1987). YAC provides the largest DNA insert capacity among all cloning vector types. Exogenous DNA fragments with sizes up to several hundred kilobase pairs or even as much as 2 Mb can be cloned into YAC vectors (Monaco and Larin, 1994). A cornerstone of the Human Genome Project (HGP) is the cloning of large chromosomal fragments using YAC vectors. However, problems are frequently observed during the use of YAC clones, including chimerics, deletions, and rearrangements. Furthermore, the isolation of YAC clones is challenging because of large sizes (Ramsay, 1994). As a result, each YAC clone must be carefully analyzed to ensure that no DNA rearrangements occur. In addition, the YAC system is established from eukaryotes and mainly used to study eukaryotic genomes, in which randomly distributed ARS sequences of 20∼30 kb, while being rarely in prokaryotic genomes (Stinchcomb et al., 1980).

#### Phage Artificial Chromosome

The first PAC vector pCYPAC1 combining the characteristics of P1-phage and F factor was developed in 1994 (Loannou et al., 1994). It can be efficiently transformed into *E. coli* via electrotransformation. Foreign DNA inserted in the PAC exhibits almost no chimerism or rearrangement. The PAC vector can carry DNA fragments of up to approximately 300 kb. The recombinant PAC can stably exist as a single copy and propagate efficiently. To facilitate the use of PAC vectors in *Streptomyces* strains, the ΦC31 *attP*-*int* elements required for chromosomal integration in *Streptomcyes* was incorporated into a pCYPAC1-derivative vector (Ioannou et al., 1996), generating so-called ESAC (*E. coli*–*Streptomyces* artificial chromosome, pESAC) vectors. Using these pESAC vectors, up to 140 kb segments of *Actinomyces* DNA can be cloned and introduced into genetically accessible *Streptomyces lividans* via protoplast transformation, stably maintaining the vector in an integrative form (Sosio et al., 2000). Using PAC vector pESAC13 (a derivative of pESAC) harboring an *oriT* site, which allows for conjugal transfer instead of time-consuming protoplast transformation, a genomic library of *Streptomyces tsukubaensis* was generated, and the entire 83.5 kb FK506 (tacrolimus) gene cluster was then identified (Jones et al., 2013). The PAC library of *Stretomyces* sp. PCS3-D2 was also constructed and analyzed *in silico*. Two clones containing 130 and 140 kb DNA inserts were identified to harbor Type I and Type III PKS (polyketide synthase) gene clusters, respectively (Bayot Custodio and Alcantara, 2019). The positive rates of recombinant clones containing DNA inserts can be greatly improved by introducing the *sacB* or *URA3* gene into PAC vectors as counter-screening markers, which can catalyze the production of toxicants in the presence of sucrose or 5-fluoroorotic acid (5-FOA), respectively (Noskov et al., 2003; Tang X. et al., 2015).

#### Bacterial Artificial Chromosome

In 1992, the first BAC vector pBAC108L was constructed based on the well-studied *E. coli* F factor. This BAC vector retained the *oriS*, *repE, parA*, and *parB* of the F factor for replication and copy number control, while also harboring a chloramphenicol resistance marker as well as the bacteriophage λ *cosN* and Pl *loxP* sites for specific cleavage by terminase and Cre enzymes, respectively. This BAC vector has been reported to carry human genomic DNA fragments approaching 300 kb (Shizuya et al., 1992). Further, it enables the cloning of large-sized DNA fragments from complex genomic sources into bacteria, where they remain stable and are easily manipulated. However, normally, only 10–50% of the clones carry DNA inserts, depending upon the batch of the vector and insert DNA used (Shizuya et al., 1992). To facilitate the screening of positive clones, the pBeloBAC11 BAC vector contains an additional component, β-galactosidase (encoded by *lacZ*), which allows clones with DNA inserts to be readily identified based on an X-gal color change. Additionally, the plndigoBAC vector displays a much faster and deeper X-gal color change as a result of a point mutation in the 3’ end of *lacZ* (Shizuya and Kouros-Mehr, 2001). Various BAC vectors, such as pStreptoBAC and pSBAC, have been extensively used for library construction with the purpose of cloning target large-sized NP BGCs (Sosio et al., 2000; Martinez et al., 2004; Miao et al., 2005; Liu et al., 2009). These BAC vectors harbor two replication origins. One is *ori* that is essential for the initiation of single-copy replication in *E. coli*, which is crucial for stability when large DNA fragments were inserted. The other is *oriV*, which can be induced to increase DNA yield.

Thus far, when compared to YAC and PAC, the BAC vectors are more commonly employed for NP BGC cloning. When the genomic sequence information of BGCs is unknown (e.g., metagenome), BAC-based library construction strategies for NP discovery are always employed. Recently, using this strategy, several large NP BGCs, such as an aminopolyol polyketide BGC over 150 kb, and a quinolidomicin BGC over 200 kb, have been successfully cloned and heterologously expressed (Zhang L. et al., 2017; Hashimoto et al., 2018; Sun et al., 2018). However, due to the low positive rates, laborious screening is necessary (Lin et al., 2020). Therefore, high-throughput screening methods have received considerable attention. Recently, the MAPLE (Microfluidic automated plasmid library enrichment) method, which combines BAC libraries with single-cell droplet microfluidic techniques for discovering functional biosynthetic pathways, was developed. Using MAPLE, a type I PKS gene cluster from an Antarctic soil metagenome was isolated and sequenced (Xu et al., 2020). In addition, when the genome sequence is available, the pSBAC vector can be inserted into the flanking regions of target BGCs within the chromosome in advance and the entire target BGCs can then be captured into pSBAC through specific restriction enzyme digestion and self-ligation. Using this method, the meridamycin (MER, ∼95 kb), tautomycetin (TMC, ∼80 kb), pikromycin (PIK, ∼60 kb), and daptomycin (DPT, ∼65 kb) BGCs have been successfully cloned (Liu et al., 2009; Nah et al., 2015; Pyeon et al., 2017; Choi et al., 2019). However, a major drawback is the problematic identification of naturally existing unique restriction enzyme recognition sites on both sides of the target BGCs. Therefore, artificial insertion of a specific DNA sequence into the genome via homologous recombination (HR) is a prerequisite, limiting the application of this method in intractable strains.

#### Fungal Artificial Chromosome

Besides bacterial strains (especially actinomycetes), fungi are also prolific producers of NPs. However, despite the abundance of available fungal genome data that encode a large number of NP BGCs, the majority of them are silent in laboratory growth conditions and most fungi are not genetically amendable. To efficiently discover fungal NPs, Bok and colleagues created a novel *Aspergillus*/*E. coli* shuttle FAC expression vector, which is modified from the BAC vector via inserting the fungal autonomously replicating element AMA1 (Bok et al., 2015). Using FAC and metabolomic scoring (MS), 56 recombinant FACs containing uncharacterized BGCs from diverse fungal species were constructed and expressed in *Aspergillus nidulans*. Finally, 15 new metabolites were discovered and assigned with confidence to their BGCs (Clevenger et al., 2017). It could be anticipated that the development of FAC will facilitate NP research of fungi in the future.

### Standardized and Orthogonal Vectors

With the rapid development of synthetic biology, standardized and orthogonal vectors, which follow uniform and modular standards, have been developed. They enable the rapid and easy exchange of modules and boost the interoperability of genetic devices among different users (Martinez-Garcia et al., 2020). However, within the field of specialized NP synthetic biology, even though there are multifarious vectors for large DNA fragment cloning, few such standard vectors have been constructed. It is well known that the size (from a few kb to more than 100 kb) of NP BGCs, the genomic GC content, and the repeat or similar sequence in the PKS or NRPS (non-ribosomal peptide synthase) genes can affect the choice of vectors for BGC cloning (Aubry et al., 2019). Thus, vectors that are flexible and adapted to various assembly methods are preferred. Recently, a suite of standardized, orthogonal integration vectors based on three site-specific integration systems (ΦBT1, ΦC31, and VWB), four antibiotic resistance genes (conferring resistance against apramycin, spectinomycin, thiostrepton, and ampicillin, respectively), and 14 promoters were constructed in order to characterize heterologous genes in *Streptomyces* species. However, these vectors were mainly used for monocistronic gene expression (Phelan et al., 2017). A set of 12 standardized and modular (three different resistance markers and four orthogonal integration systems) vectors based on model SEVA plasmids were designed to allow for the assembly of NP BGCs through various cloning methods in *Streptomyces* species (Aubry et al., 2019). In these vectors, the FLP (flippase) recombination system was also incorporated for the recycling of antibiotic markers and for reducing unwanted homologous recombination when several vectors are used simultaneously (Aubry et al., 2019). It can be expected that through the modularization and orthogonalization of key vector elements, including orthogonal integration systems, origins of replication, antibiotic selection markers, and a variety of cargoes with specific applications, a suitable vector can be quickly designed to efficiently assemble or clone large DNA fragments. It is worth to note that so far, many laboratories have designed and constructed a large number of multifarious vectors according to their own needs. To further promote NP research, laboratories should make their vectors freely available to other research groups.

## Assembly/Cloning Methods

High fidelity, effective and seamless assembly of large DNA fragments and appropriate vectors is the pivotal step for obtaining entire NP BGCs for heterologous expression. With the rapid development of synthetic biology, various DNA cloning and assembly methods have been established and successfully utilized for cloning NP BGCs. Depending on the experimental setting, assembly methods can be divided into two categories: *in vitro* and *in vivo* DNA assembly (Juhas and Ajioka, 2017; Li et al., 2017; Aubry et al., 2019; Kang and Kim, 2021).

*In vitro* cloning and assembly approaches include three main types: (1) restriction enzyme-mediated methods, e.g., BioBrick, Golden Gate, and MASTER ligation (Engler et al., 2009; Chen et al., 2013); (2) recombination-based assembly methods, such as Gibson assembly (Gibson et al., 2009), ligase cycling reaction (LCR) (Schlichting et al., 2019), direct pathway cloning (DiPaC) (Greunke et al., 2018), and DNA assembly methods based on the use of site-specific integrases (e.g., ΦC31, ΦBT1) (Li et al., 2017); (3) enzyme-independent DNA assembly, including enzyme-free cloning (EFC) and twin primer non-enzymatic DNA assembly (TPA) (Liang et al., 2017). NP BGCs can also be directly captured using several *in vivo* methods, including the use of Red/ET system-mediated cloning tools, such as linear-linear homologous recombination (LLHR) or linear-circular-homologous recombination (LCHR) (Fu et al., 2012), exonuclease combined with Red/ET (ExoCET) (Wang et al., 2018), transformation-associated recombination (TAR) cloning method based on the natural recombination capability of *S. cerevisiae* (Noskov et al., 2011; Kouprina et al., 2020), and site-specific recombination (SSR) system-based tools (Du et al., 2015; Gao et al., 2020).

### *In vitro* DNA Assembly Approaches

#### Restriction Enzyme-Mediated Methods

**FIGURE 2:**
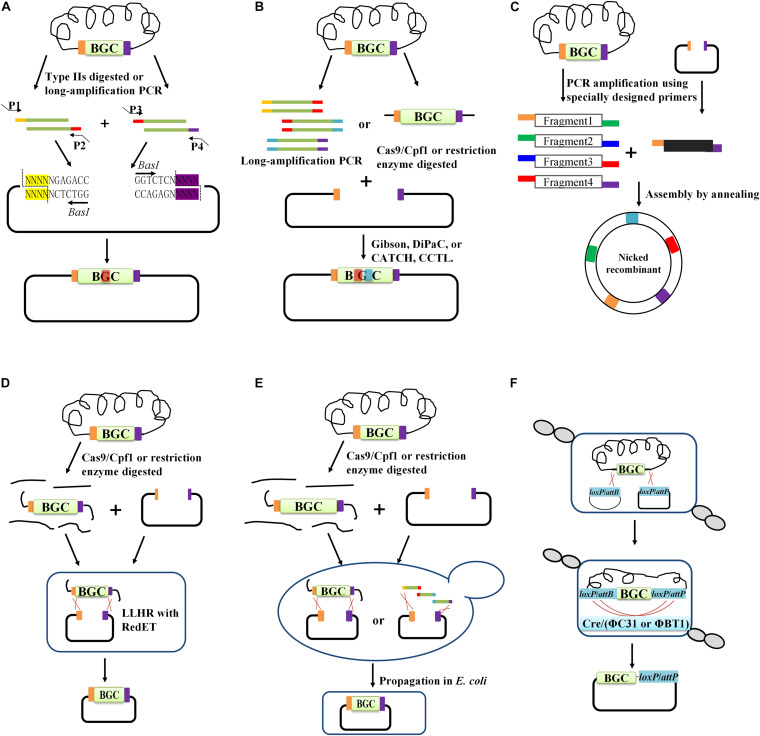
The main cloning methods for BGC capturing. *In vitro* DNA assembly method including: **(A)** restriction enzyme-mediated digestion and ligation (e.g., Golden gate), **(B)** recombination-based assembly methods (e.g., Gibson assembly, DiPaC, CATCH, and CCTL), **(C)** enzyme-independent DNA assemble method. *In vivo* assembly approaches including: **(D)** phage-recombinase-mediated HR in *E. coli*, **(E)** TAR cloning in yeast. **(F)** Site-specific integrase (e.g., Cre/loxP, ΦC31, or ΦBT1) mediated cloning.

The classic method for DNA assembly is via the use of enzymes for the cutting and ligation of DNA fragments and vectors. However, these will leave scars at the restriction site. To address this problem, type IIs restriction enzymes (e.g., *Bbs*I, *Bsa*I, and *Bpi*I), which cut outside of the recognition sites and generate single-stranded DNA overhangs, are employed. The DNA overhangs can be appropriately designed to guide the corresponding DNA fragments for ligation in a designated order. This method was named Golden Gate (Figure 2A), which reflects the concept of modular assembly (Mitchell et al., 2015). Recently, it was employed for refactoring carotenoid biosynthetic pathways. In particular, each biosynthetic gene equipped with different promoters and terminators was assembled, resulting in various expression cassettes. A library containing 96 combinatorial refactored carotenoid pathways was then successfully generated by assembling these cassettes (Ren et al., 2017). Based on type IIs restriction enzymes, a Golden Gate shuffling method was developed, which can achieve the assembly of at least nine DNA fragments in a single step with high efficiency (90%) (Engler et al., 2009). A similar method named MASTER (methylation-assisted tailorable ends rational) ligation based on MspJI, a specific type IIs endonuclease, was also developed for sequence-independent hierarchical DNA assembly. Using the MspJI-mediated method, the blue-colored antibiotic actinorhodin (ACT) BGC (29 kb) from *Streptomyces coelicolor* was successfully assembled and expressed in a fast-growing *Streptomyces* sp. (Chen et al., 2013). To be appropriate for Golden Gate cloning, special care should be taken to ensure that the type IIs restriction site is present in opposite orientation at the ends of the vector and DNA fragments but absent in internal sequences (Marillonnet and Grutzner, 2020). However, type IIs enzymes are relatively rare, and thus few options are available. Usually, internal type IIs restriction sites should be removed by silent mutations. In addition, the number of DNA fragments that can be simultaneously assembled is still limited (Schmid-Burgk et al., 2013; Marillonnet and Grutzner, 2020).

#### Recombination-Based Assembly Methods

Although traditional restriction cutting and ligation methods are still widely used, their low efficiency and enzyme site-dependence do not meet the increasing demand for assembling large DNA fragments. Thus, recombination-based assembly methods (Figure 2B) based on the existence of short homologous regions at the extremities of DNA fragments and vectors are attracting more attention. These methods include ligation-independent cloning (LIC) (Aslanidis and de Jong, 1990), sequence and ligation-independent cloning (SLiC) (Li and Elledge, 2012), seamless ligation cloning extract (SliCE) (Zhang Y. et al., 2012), circular polymerase extension cloning (CPEC) (Quan and Tian, 2014), Gibson assembly, and Cas9-associated targeting of chromosome segments (CATCH) (Jiang et al., 2015) and so on. Recombination-based DNA assembly usually employs one to three enzymes in the *in vitro* reaction, wherein DNA polymerase, exonuclease, and ligase are the most commonly used. Hereafter, we provide a brief introduction to the above-mentioned assembly methods.

Ligation-independent cloning mediates the assembly between a DNA fragment and a PCR-amplified vector with a 12-nt tail complementary to the DNA fragment’s end. It does not require the use of restriction enzymes and T4 DNA ligase. The 3′-terminal sequence can be removed via the (3′→5′) exonuclease activity of T4 DNA polymerase, leading to DNA fragments with a 5′-overhang (10–12 nt in length), which results in annealing and circularization between vector molecules and DNA fragments mediated by the 10–12-nt cohesive ends (Aslanidis and de Jong, 1990). Based on this method, SLIC was developed, which can achieve the assembly of multiple DNA fragments in a single reaction by combining *in vitro* HR and single-strand annealing. SLIC is more efficient at very low DNA concentrations, especially in the presence of the HR protein RecA (Li and Elledge, 2007). SLiCE is a highly cost-effective method, which utilizes cell extracts from *E. coli* with overexpression of the λ phage Red recombination system for DNA assembly *in vitro*. This method provides an effective strategy for directional seamless DNA cloning from BAC or complex genomes (Zhang Y. et al., 2012).

The Gibson assembly method uses three commercial enzymes (T5 exonuclease, Phusion DNA polymerase, and Taq DNA ligase) for the assembly of DNA fragments with short homologous ends *in vitro*. Unlike the T4 DNA polymerase in LIC, which produces a 5’-overhang, T5 exonuclease chews back the homologous ends to generate 3’-overhangs, which anneal to each other, followed by Phusion DNA polymerase and Taq DNA ligase, which fill the gap and covalently link the fragments, respectively. However, Gibson assembly cannot be efficiently employed for the assembly of DNA fragments with high GC content due to high vector self-ligation background. Recently, a modified Gibson assembly method was developed by adding a pair of universal terminal overhangs with high AT content (21 bp) to the ends of the BAC vector, greatly reducing vector self-ligation (Li L. et al., 2015). Using this method, a 67 kb pristinamycin II (PII) BGC from *Streptomyces pristinaespiralis* was hierarchically assembled from 15 PCR-amplified fragments (Li L. et al., 2015). In addition, a T5 exonuclease-mediated DNA assembly (TEDA) method was established, in which homologous ends were treated with T5 exonuclease alone. After annealing, the reaction sample was transformed into *E. coli* to repair the gap and form a phosphodiester bond to link the fragment and vector with the endogenous DNA repair enzymes. The results indicated that the cloning efficiency of TEDA was higher than that of the traditional Gibson assembly tool (Xia et al., 2019). The development of CRISPR technology has greatly promoted recombination-mediated DNA assembly. A novel DNA assembly method named CATCH was developed by combining the *in vitro* CRISPR/Cas9 endonuclease-mediated genome treatment and Gibson assembly, which could achieve the direct cloning of large bacterial genomic segments (up to 100 kb) (Jiang et al., 2015). Using this tool, the 78-kb bacillaene BGC from *Bacillus subtilis* was cloned into a BAC vector at a ∼12% positive rate. In addition, the 36 kb jadomycin BGC from *Streptomyces venezuelae* and the 32 kb chlortetracycline BGC from *Streptomyces aureofaciens*, were also successfully captured with a ∼90% positive rate, highlighting the versatility of CATCH for cloning large BGCs (Jiang et al., 2015). It should be noted that these recombination-based methods (e.g., Gibson assembly, CATCH) might be inefficient when homologous regions of the fragment extremities have complicated DNA sequences, such as secondary hairpin structure formation or high GC content (Casini et al., 2014; Li L. et al., 2015).

Unlike Cas9 which introduces double-strand breaks (DSBs) and produces blunt ends, Cpf1 (Cas12a) cleaves target DNA and produces sticky overhangs, which makes Cpf1 an alternative tool for DNA assembly *in vitro* (Lei et al., 2017). Recently, a method named CCTL (Cpf1-assisted Cutting and Taq DNA ligase mediated Ligation) was developed, with the 8-nt sticky end produced by Cpf1 cleavage for ligation instead of homologous sequences in recombination-based methods, which therefore make the CCTL is suitable for cloning complicated DNA sequences (Lei et al., 2017). However, the requirement of specific PAM limits its application. To address this limitation, the PAM specificity of Cas12a was expanded via specific structure-guided mutagenesis and two engineered Cas12a variant EP15 and EP16 were obtained, which increased the targeting range by fourfold. Based on this modified Cas12a, the iCOPE (improved Cas12a-assisted one-pot DNA Editing) method was developed, which can avoid many of the DNA sequence constraints (Wang et al., 2019).

In addition to the methods described above, various *in vitro* novel DNA assembly methods for BGCs cloning have been designed and established. These include the assembly of fragment ends after PCR (AFEAP cloning) (Zeng et al., 2017), versatile genetic assembly system (VEGAS) (Mitchell et al., 2015), uracil-specific excision reagent cloning (USER) (Salomonsen et al., 2014), LCR (de Kok et al., 2014), DNA assembly with thermostable exonuclease and ligase (DATEL) (Jin et al., 2016), overlap extension PCR and recombination (OEPR Cloning) (Liu C. J. et al., 2017), direct pathway cloning (DiPaC) (Greunke et al., 2018), as well as DiPaC combination of SLIC cloning (D’Agostino and Gulder, 2018), which provide alternatives for large fragment cloning under different experimental conditions.

#### Enzyme-Independent DNA Assembly

Enzyme-mediated DNA assembly methods are efficient and straightforward. As mentioned above, Golden Gate assembly is robust and suitable for assembling over 15 DNA fragments with high efficiency and fidelity. However, due to limited commercially available Type IIs endonucleases, it is not always possible to find suitable restriction enzymes that avoid the naturally occurring Type IIs sites within BGCs (Liang et al., 2017). Therefore, additional efforts are needed to modify the sequences of BGCs in order to eliminate the undesired cut sites. Gibson assembly is versatile, but its efficiency and fidelity drop sharply when the number of fragments is more than four. Furthermore, essential components such as promoters, ribosomal binding sites, and terminators are notoriously difficult for Gibson assembly because of their secondary structures (Liang et al., 2017). Enzyme-independent DNA assembly methods can realize DNA assembly without enzymes, which saves costs and is especially suitable for high-throughput settings. These approaches mainly include enzyme-free cloning (EFC), polymerase incomplete primer extension (PIPE), and Twin primer non-enzymatic DNA assembly (TPA).

A highly efficient EFC procedure for DNA assembly was previously established, utilizing tailed PCR primer sets to generate complementary staggered overhangs on both fragments and vectors via a denaturation-hybridization reaction (Tillett and Neilan, 1999). This approach enables directional cloning in a ligase-free manner. Therefore, it is not constrained by the requirement for appropriate enzyme sites. However, this method is mainly used for the assembly of two DNA fragments, and its efficiency is low. TPA for the efficient assembly of multiple PCR fragments was recently developed (Figure 2C), allowing for the successful construction of a 31 kb plasmid harboring an *n*-butanol production pathway (∼26 kb) from five fragments with ∼50% fidelity (Liang et al., 2017). TPA cloning is also seamless and sequence-independent, and its performance rivals even the best *in vitro* assembly methods. Although these enzyme-free cloning tools provide a number of advantages over other cloning strategies, they still have limitations. For example, these methods usually require a number of specially designed primers, and the assembly capability as well as fidelity drop sharply with increasing fragment size (Tillett and Neilan, 1999; Yuan et al., 2016; Liang et al., 2017; Richter et al., 2019).

### *In vivo* Assembly Approaches

*In vitro* assembly methods provide flexible cloning of DNA fragments, wherein the DNA fragments can be produced through multiple rounds of PCR or direct chemical synthesis. However, random mutations cannot be entirely ruled out. In addition, incorrect pairing of DNA fragments during assembly may also cause unanticipated mutations, especially in the PKS or NRPS genes, which contain numerous repeat sequences. *In vivo* DNA cloning methods for direct capture of the target DNA fragment, which are based on the strong homologous recombination ability of *E. coli* expressing the Red/ET system or that of yeast, have previously been developed. They represent an alternative strategy for BGC cloning. These methods mainly include phage recombinase-mediated homologous recombination cloning in *E. coli* such as LLHR (Figure 2D), transformation-associated recombination-mediated cloning (TAR) in yeast (Figure 2E) and site-specific recombination (SSR)-mediated cloning in *Streptomyces* (Figure 2F; Li et al., 2017; Abbasi et al., 2020; Kang and Kim, 2021).

#### Phage-Recombinase-Mediated HR in *E. coli*

The endogenous HR system in *E. coli* is mainly mediated by the chromosome-encoded recombinases RecA/RecBCD (Abbasi et al., 2020). Many cloning strategies based on the endogenous HR have been created, such as *in vivo* cloning (IVC), in which PCR products containing terminal sequences identical to the two terminals of the linearized vector were co-transfected into *E. coli* to incorporate PCR fragments into the vector via the high HR ability of *E. coli* (Oliner et al., 1993). However, due to its strong exonuclease activity, the RecBCD complex can rapidly degrade exogenous linear DNA molecules. The PCR products and linear vector can be introduced and stably maintained only in RecBCD-deficient *E. coli* strains (Abbasi et al., 2020). In addition, RecA-dependent recombination requires a much longer homologous region (approximately 500 bp). To develop a more efficient and reliable HR system in *E. coli*, Red/ET recombineering was developed, which depends on phage-recombinases, either RecE/RecT from the Rac prophage or Redα/Redβ from the λ phage (Zhang et al., 1998). RecE and Redα are 5′→3′ ATP-independent exonucleases, while RecT and Redβ are DNA annealing proteins. Another protein, Redγ, identified only in the λ phage, was found to significantly promote the recombination efficiency of Redα/Redβ. It was later identified as an inhibitor of the RecB subunit of RecBCD. This protein protects linear DNA from degradation by endogenous nucleases (Abbasi et al., 2020).

Red/ET recombineering has been established as an efficient *in vivo* homologous recombination strategy for *E. coli* (Wang et al., 2016). This technology was first used to reconstitute an entire 43 kb myxochromide BGC from two overlapping cosmids (Wenzel et al., 2005). Subsequently, it has been widely applied for the cloning of a variety of NP BGCs ranging from 11 to 106 kb from different microbes, including *Streptomyces, Sorangium*, and *Cystobacter* (Binz et al., 2008; Lesic and Rahme, 2008; Wang et al., 2018). In these cases, the reconstitution process was mediated by very short homologous regions (usually 40–50 bp) between a replicative circular vector and a linear DNA molecule, and was therefore termed “linear-circular homologous recombination (LCHR).” However, the approach utilizing Redαβ or the truncated version of RecET is inefficient at mediating homologous recombination between two linear DNA molecules, which hampers its use for direct cloning of target BGCs (Fu et al., 2012).

Fu et al. (2012) discovered that full-length RecE along with RecT considerably increased the efficiency of recombination between two linear DNA molecules (a linearized target DNA fragment and a PCR-amplified linear vector backbone flanked with homology arms to the target DNA). Using this LLHR (linear–linear homologous recombination) approach, ten large NRPS and PKS BGCs (with sizes from 10 to 37 kb) from the genomic DNA of *Photorhabdus luminescens* were directly cloned into linear expression vectors in a one-step recombination event. However, they failed to direct clone the intact 106 kb salinomycin gene cluster from the genome of *Streptomyces albus* using LLHR. Finally, the group successfully cloned three fragments of salinomycin BGC using LLHR separately and assembled them into a complete one (Yin et al., 2015).

To improve the performance for direct cloning of large-sized (>50 kb) DNA segments from complex genomes such as mammalian genomes, which are three orders of magnitude larger than bacterial genomes, exonuclease (*in vitro*) combined with RecET recombination (*in vivo*) (ExoCET) was developed (Wang et al., 2018). For the *in vitro* assembly, several exonucleases including T4 polymerase (T4 pol), T5 exonuclease, T7 exonuclease, DNA polymerase I Klenow fragment, T7 DNA polymerase, λ exonuclease, Exonuclease III, and Phusion DNA polymerase, were tested. The 3’ exonuclease activity of T4 polymerase was selected due to it having the highest efficiency and fidelity (Wang et al., 2018). After exonuclease chew-back, the target DNA fragment and the vector were annealed together via the homology arm (about 80 bp) and were then transformed into *E. coli* for *in vivo* HR via Red/ET. This concerted action of T4 pol and Red/ET is believed to be more proficient for the direct cloning of long DNA regions than either T4 pol or Red/ET alone (Wang et al., 2018). ExoCET is generally applicable to a broader range of direct cloning with respect to size (up to 106 kb) and genome complexity (Wang et al., 2018). It should be noted that, in order to ensure a high efficiency for the LLHR-mediated cloning method, genomic DNA must be cleaved by unique restriction enzymes near the 5’ and 3’ ends of target BGCs. However, it is not always easy to find appropriate restriction enzyme cutting sites. With the advent of the programmable CRISPR/Cas9 system, which is able to recognize and cut DNA sequences near target BGCs to easily release linear DNA fragments, this limitation could be overcome (Lee et al., 2015; Wang et al., 2018). With improved Red/ET technology and rapidly growing microbial genome sequence data in public databases, a variety of complete NP BGCs have been cloned directly from microbial genomic DNA via LLHR (Table 1).

**TABLE 1:** Examples of BGCs cloned by Red/ET recombination (from 2015 to present).

Natural products	NP Type	Biological activity	Native host	Size (kb)	Cloning efficiency^a^	Heterologous host for NP expression	Production	References
Streptoketides	Type-II PKS	Anti-HIV	*Streptomyces* sp. Tü6314	22	8.3% (1/12)	*S. coelicolor*	NR	Qian et al., 2020
Spinosad	Type-I PKS	Insecticide	*Saccharopolyspora spinosa*	79	NR	*S. albus*	1116 μg/L	Song et al., 2019
Chuangxinmycin	Indole alkaloid	Antibacterial	*Actinoplanes tsinanensis*	11	NR	*S. coelicolor*	NR	Xu et al., 2018
Syringolin	NRPS	Antitumor	*Pseudomonas syringae*	22	NR	*S. coelicolor; S. lividans*	NR	Huang et al., 2018
Microcystin	Hybrid PKS-NRPS	Cyanotoxins	*Microcystis aeruginosa*	55	NR	*E. coli*	65 μg/L	Liu T. et al., 2017
Novobiocin	NRPS	DNA gyrase inhibitor	*Streptomyces spheroides*	20	NR	*S. coelicolor*	40 μg/L	Basitta et al., 2017
Disorazol	Type-I PKS	Inhibit cancer cell proliferation	*Sorangium cellulosum*	58	NR	*Myxococcus xanthus*	1 mg/L	Tu et al., 2016
Edeine	NRPS	Antimicrobial	*Brevibacillus brevis*	48	8.3% (2/24)	*B. subtilis*	ND	Liu et al., 2016
Bacillomycin	NRPS	Antifungal	*Bacillus amyloliquefaciens*	37	12.5% (3/24)	*B. subtilis*	NR	Liu et al., 2016
Plu3535-Plu3532	NRPS	NR	*Photorhabdus Luminescens*	38	60% (6/10)	**–**	–	Wang et al., 2016
Plu2670	NRPS	NR	*P. luminescens*	53	83.3% (10/12)	**–**	–	Wang et al., 2018
Salinomycin	Type-I PKS	Anti-cancer	*S. albus*	106	4.2% (1/24)	*S. coelicolor*	NR	Wang et al., 2018
Sevadicin	NRPS	Against *Bacillus Megaterium*	*Paenibacillus larvae*	12	NR	*E. coli*	NR	Tang Y. et al., 2015

*^*a*^Numbers in brackets represent the ratio of the number of positive clones relative to the total clones.*

*NR, not reported; ND, not detected.*

#### TAR Cloning of NP BGCs

The assembly of two DNA molecules containing homologous sequences via recombination in yeast was first demonstrated by Kunes et al. (1985). A couple of years later, a convenient method for plasmid construction using this *in vivo* bimolecular recombination reaction was developed (Ma et al., 1987). Motivated by this method, a transformation-associated recombination (TAR) strategy in yeast based on this approach was later introduced, allowing for the selective isolation of large genomic regions from complex genomic DNA (Larionov et al., 1997; Noskov et al., 2002).

Transformation-associated recombination was initially been used to isolate large regions of mammalian genomic DNA in the 1990s (Larionov et al., 1997). The propagation of TAR-generated DNA constructs depends on ARS-like sequences, which can function as an origin of replication in yeast. The ARS sequences are frequently and randomly distributed throughout all eukaryotic genomes per 20–30 kb on average (Stinchcomb et al., 1980). Chromosomal regions with high G + C content are poor in ARS-like sequences, and ARS frequency might be reduced in prokaryotic genomes, which precludes their isolation via the standard TAR method. Noskov et al. (2003) inserted ARS into the TAR vector, using HIS3 as a positive selection marker and URA3 as a negative marker. The modified TAR cloning system enables the isolation of genomic regions lacking yeast ARS-like sequences (e.g., bacterial genome DNA) and eliminates the high vector recircularization background caused by end-joining during yeast transformation (Noskov et al., 2003). This modified TAR cloning method was further extended to capture microbial NP BGCs by constructing the yeast-*E. coli*–*Streptomyces* tri-shuttle vector pTARa. Using pTARa, multiple BGCs were directly cloned or reassembled from environmental DNA (eDNA) libraries (Kim et al., 2010). In contrast to pTARa that harbors *oriV*, pCAP01, a novel capture vector equipped with a pUC *ori*, can maintain multiple copies without induction and remained stable even when carrying > 50 kb inserts (Yamanaka et al., 2012, 2014). Using the pCAP101 vector, a 67 kb silent NRPS BGC responsible for the biosynthesis of taromycin from the marine actinomycete *Saccharomonospora* sp. CNQ-490 was successfully captured and activated in *S. coelicolor* M1146 (Yamanaka et al., 2014). However, the construction process of pCAP01-based capture plasmids is tedious and time-consuming. It involves the assembly of a pair of 1-kb capture arms into pCAP01, overlapping with the flanking regions of target BGCs. Larson et al. (2017) streamlined this procedure by employing a fully synthetic 360 bp capture arm, which reduced the duration of the cloning process and opened the door for high-throughput applications. Using this modified TAR method, a 54 kb cosmomycin BGC from *Streptomyces* sp. CNT-302 was successfully captured (Larson et al., 2017). The range of heterologous hosts compatible with the TAR platform was expanded to the Gram-positive *Bacillus subtilis* with low G + C content by replacing the high G + C content *Streptomyces* element in pCAP01 with the *Bacillus* element (Bourgouin et al., 1990) to yield the yeast-*E. coli*-*B. subtilis* tri-shuttle vector pCAPB1. Using pCAPB1, the surfactin BGC was successfully cloned from *B. subtilis* 1779 (Li Y. et al., 2015). Later, a TAR vector pCAP05 was constructed by introducing an RK2 replicon. It replicates at a low copy number in a wide range of Gram-negative bacteria via the *oriV* and *trfA* gene, which determine host range and copy number (Scott et al., 2003; Zhang J. J. et al., 2017). Using pCAP05, the violacein BGC (∼8 kb) from marine bacterium *Pseudoalteromonas luteoviolacea* was cloned and expressed in *Pseudomonas putida* and *Agrobacterium tumefaciens* (Zhang J. J. et al., 2017). Overall, TAR has been widely employed for BGC cloning, leading to the identification of many novel NPs (Alberti et al., 2019; Kouprina and Larionov, 2019; Table 2).

**TABLE 2:** Examples of BGCs cloned by TAR cloning (from 2015 to present).

Natural products	NP type	Biological activity	Native hosts	Size (kb)	Cloning efficiency^a^	Heterologous hosts for NP expression	Production	References
Bostrycoidin	Type-I PKS	Pigment	*Fusarium solani*	–	NR	*S. cerevisiae*	2.2 mg/L	Pedersen et al., 2020
Scleric acid	NRPS	Against *M. tuberculosis*	*Streptomyces sclerotialus*	33	NR	*S. albus*	NR	Alberti et al., 2019
Cadasides	NRPS	Antimicrobial	Soil Metagenome	66	10% (1/10)	*S. albus*	NR	Wu et al., 2019
Plipastatin	NRPS	Against fungi	*B. amyloliquefaciens*	40	16.7% (1/6)	*B. subtilis*	1182.5 mg/L	Hu et al., 2018
Malacidins	NRPS	Calcium-dependent antibacterial	Soil Metagenome	67	NR	*S. albus*	NR	Hover et al., 2018
Demethyl chlortetracycline	Type-II PKS	Antibacterial	*Streptomyces aureofaciens*	44	2.4% (4/164)	*S. aureofaciens*	655 mg/L	Wu et al., 2017
Pristinamycin	Hybrid PKS/NRPS	Anti-MRSA	*S. pristinaespiralis*	39	NR	*S. pristinaespiralis*	132 mg/L	Meng et al., 2017
Cosmomycin	Type-II PKS	Antitumor	*Streptomyces* sp. CNT-302	54	1.5% (3/200)	*S. coelicolor*	4 mg/L	Larson et al., 2017
Grecocycline	Type-II PKS	NR	*Streptomyces* sp. Acta 1362	36	23%	*S. albus*	26 mg/L	Bilyk et al., 2016
Salinamide	NRPS	Anti-inflammatory and antibacterial	*Streptomyces* sp. CNB-091	48	NR	*S. coelicolor*	NR	Ray et al., 2016
Ammosamide	Alkaloid	NR	*Streptomyces* sp. CNR-698	38	NR	*S. coelicolor*	134 mg/L	Jordan and Moore, 2016
Thiotetronic Acid	Hybrid PKS/NRPS	Antibacterial	*Salinispora* Pan-genome	22	66.7% (8/12)	*S. coelicolor*	NR	Tang X. et al., 2015
Alterochromide	NRPS	Antibacterial	*Pseudoalteromonas piscicida*	34	NR	*E. coli*	60-fold less than that in native host	Ross et al., 2015
Surfactin	NRPS	Antibacterial	*B. subtilis* 1779	38	NR	*B. subtilis* JH642	100-fold less than that in native host	Li Y. et al., 2015
Enterocin	Type-II PKS	Bacteriostatic	*Salinispora pacifica*	21	NR	*S. coelicolor*	NR	Bonet et al., 2015

*^*a*^Numbers in brackets represent the ratio of the number of positive clones to the total clones.*

*NR, not reported.*

Although TAR cloning can be used to directly clone NP BGCs of interest, the method exhibits very low cloning efficiency (0.5–2%) due to vector recircularization via end joining in yeast, which leads to time-consuming screening of hundreds of clones. Thus, two different strategies have been introduced to increase the positive rates (Lee et al., 2015; Tang X. et al., 2015). The first one is to use a counter-selection marker for colony selection. Tang X. et al. (2015) introduced the *URA3* gene under a strong pADH1promoter into pCAP01 in order to generate pCAP03, which allows for convenient screening against recircularization in the presence of 5-FOA. Using pCAP03, a 26 kb thiolactomycin BGC from *Salinispora pacifica* was captured at a positive rate of 75%, and a 33 kb genome locus containing the thiotetroamide BGC (∼29 kb) was cloned at a positive rate of 20% (Tang X. et al., 2015). The second strategy is to use the RNA-guided Cas9 endonuclease to cleave chromosomal DNA (Lee et al., 2015). Homologous recombination has been reported as more efficient when the linearized capturing vector hooks (homology arms) are located closer to the ends of the target DNA sequences (Kouprina et al., 2006). Although unique restriction enzymes can be theoretically obtained to cleave near the 5’ and 3’ ends of target DNA, it is always challenging to find suitable cutting sites. The programmable CRISPR/Cas9 system was used to precisely cleave both sides of the target DNA, significantly improving TAR cloning efficiency by up to 32% (Lee et al., 2015). Currently, capturing target chromosomal regions requires the screening of less than a dozen transformants. It is conceivable that TAR cloning, combined with a counter-selection marker and the CRISPR/Cas9 system, will further accelerate the direct cloning of microbial NP BGCs. So far, TAR cloning is the only available method for selectively capture chromosomal segments up to 300 kb from complex genomes (Kouprina and Larionov, 2016). Collective examples for the direct cloning of NP BGCs by TAR are summarized in Table 2.

#### Site-Specific Integrase-Mediated Cloning

In addition to DNA cloning and assembly methods based on homologous recombination in *E. coli* or yeast, there are other *in vivo* cloning systems based on site-specific recombination (SSR), which consist of a specialized recombinase and its target sites. There are two evolutionarily distinct site-specific recombinases with different recombination mechanisms, including tyrosine recombinases (e.g., Cre recombinase) and serine integrases (e.g., ΦC31 and ΦBT1 integrase) (Fogg et al., 2014).

Generally, bacteriophage-derived serine integrases bind to specific 40–60 bp DNA sites (so-called attachment sites derived from the phage *attP* and cognate bacterial chromosome *attB*) and bring these sites together, cut and then rejoin the sites to yield the recombinant product (Grindley et al., 2006). Site-specific serine integration systems have been mainly used to integrate foreign DNA constructs into the *attB* site of prokaryotes, eukaryotes, or archaea chromosomes for the production of stable engineered strains. Integrases are capable of promoting efficient genomic integration of large NP BGCs (>100 kb) via *attP* × *attB* unidirectional recombination (Myronovskyi and Luzhetskyy, 2013). Based on this SSR system, a novel strategy for cloning large BGCs was devised in *Streptomyces* based on the ΦBT1 integrase (Du et al., 2015). First, the paired ΦBT1 integration sites *attB*/*attP* and the replicative plasmid pKC1139 are individually introduced on either side of the target BGC via two single crossover recombination events. Thereafter, the ΦBT1 recombinase is expressed, which mediates the cleavage of the two paired integration sites, resulting in circularization of the target BGC in pKC1139. Recombinant clones containing the target BGC are then extracted and transferred into *E. coli* for recovery. Using this strategy, the actinorhodin BGC (25 kb) from *S. coelicolor*, the napsamycin BGC (45 kb), and the daptomycin BGC (157 kb) from *Streptomyces roseosporus* were successfully isolated with high efficiency greater than 80% (Du et al., 2015). The entire 34 kb neomycin BGC from *Streptomyces fradiae* CGMCC 4.576 was similarly cloned using the ΦBT1 integration system (Zheng et al., 2019).

The Cre enzyme, as well as Flp and Dre recombinases, belongs to the tyrosine recombinase family. Cre recombinase can specifically and efficiently catalyze recombination between two specific 34-bp sites called *loxP*. The Cre/*loxP* system is effective in both bacterial and eukaryotic cells. Cre-mediated recombination results in the excision of the intervening DNA segment and produces a circular DNA molecule if two *loxP* sites in the DNA strand are in the same orientation. Therefore, when a cloning vector backbone is included in the intervening DNA, the circularized DNA molecule can replicate as a plasmid. Using this “Cre/*loxP* plus BAC” strategy, the 32 kb T3SS (type 3 secretion system) gene cluster from *Photorhabdus luminescens* and the 78 kb siderophore BGC from *A. tumefaciens* were successfully cloned (Hu et al., 2016).

However, as described above, SSR-mediated cloning methods require the initial integration of specific sites into the chromosome in advance. Therefore, they cannot be employed in difficult-to-manipulate organisms. Recently, a robust BGC cloning method named CAPTURE (Cas12a-assisted precise targeted cloning using *in vivo* Cre-*loxP* recombination) was developed by combining *in vitro* Cas12a-based treatment of genome and *in vivo* Cre-*loxp* recombination. This method could achieve direct NP BGC cloning with high efficiency (Enghiad et al., 2021). The microbial genome was purified and digested by the Cas12 protein to release the target BGC and then mixed with two PCR-amplified vector elements in a T4 DNA polymerase exo + fill-in DNA assembly reaction to join the three fragments into a linear DNA product. Finally, the linear DNA assembly products were transformed into *E. coli* expressing Cre recombinase for *in vivo* Cre-*loxp* circularization. This method avoids pre-insertion sites at both ends of the BGCs in the genome that are difficult to manipulate genetically. In addition, each PCR amplified vector element only contains one *loxP* site and does not carry the selection marker and the origin of replication, which could eliminate vector recircularization. Using CAPTURE, 47 NP BGCs ranging from 10 to 113 kb from both *Actinomyces* and *Bacilli* were directly cloned with up to 100% efficiency. Heterologous expression of the cloned BGCs led to the discovery of 15 previously uncharacterized NPs (Enghiad et al., 2021).

## Concluding Remarks

Exploring new antibiotics to combat against emerging drug resistance as well as the identification of new lead drugs for the treatment of various diseases are of utmost necessity. Thus, mining of NPs will continue to play an indispensable role in the drug discovery field. Traditionally, NP BGCs of interest are often cloned by construction of genomic DNA libraries using cosmids, fosmids, or artificial chromosomes. These methods are sequence-independent and have been proven to be efficient for cloning NP BGCs. However, these conventional methods are not suitable for the large-scale and high-throughput discovery of novel natural agents due to the requirement of extensive screening. With the availability of an increasing number of bacterial genome sequences and progress in genetic manipulation techniques, a variety of approaches for the direct cloning of large-sized BGCs from chromosomes have been developed. After carefully preparing high-quality large DNA fragments harboring putative BGCs and selecting appropriate vectors, these BGCs can be assembled or directly cloned with high efficiency *in vitro* or *in vivo* (Tables 1–3). Upon cloning, BGCs can be introduced into suitable microbial hosts for heterologous expression and subsequent identification of the corresponding products.

**TABLE 3:** Examples of BGCs cloned via *in vitro* assembly (from 2015 to present).

Natural products	NP type	Biological activity	Native host	Size (kb)	Cloning efficiency^a^	Heterologous host for NP expression	Production	References
Bacillaene	Hybrid PKS-NRPS	Inhibit prokaryotic growth	*Bacillus subtilis*	78	11.8% (12/102)	NR	NR	Jiang et al., 2015
Jadomycin	Type-II PKS	Anti-bacterial	*Streptomyces venezuelae*	36	89.9% (179/199)	NR	NR	Jiang et al., 2015
Chlortetracycline	Type-II PKS	Anti-bacterial	*S. aureofaciens*	32	90.1% (212/234)	NR	NR	Jiang et al., 2015
Salinomycin	Type-I PKS	Anti-cancer	*S. albus*	200	46.7%	NR	NR	Zeng et al., 2017
Fontizine A	Phenazine	NR	*S. fonticola*	9.5	NR	*E. coli*	∼0.4 mg/L	Greunke et al., 2018
Anabaenopeptin	NRPS	Inhibit protease	*Nostoc punctiforme*	29.2	NR	*E. coli*	>100-fold higher that in native host	Greunke et al., 2018
Erythromycin	Type-I PKS	Anti-bacterial	*S. erythraea*	54.6	NR	*S. coelicolor*	NR	Greunke et al., 2018
Hapalosin	Hybrid PKS-NRPS	reverse multiple drug resistance	*Fischerella* sp. PCC 9431	23	NR	*E. coli*	NR	D’Agostino and Gulder, 2018
Zeaxanthin (containing xylose, cellobiose using pathway)	Carotenoid pigment	–	*–*	44	71%	Yeast	0.93 mg/L	Yuan et al., 2016

*^*a*^Numbers in brackets represent the ratio of the number of positive clones to the total clones.*

*NR, not report.*

The aforementioned methods differ in both mechanism and cloning scale, providing effective means to meet different needs. The development of *in vivo*, *in vitro*, or even *in vivo/in vitro* hybrid strategies, especially those employing Cas9 or Cas12a cleavage, has greatly facilitated the cloning or assembly of microbial NP BGCs. It is therefore expected that these methodologies will greatly improve genome mining efforts that precede the discovery of novel compounds. However, to our knowledge, a universal approach suitable for all experimental situations is still lacking. Therefore, the combination of different cloning approaches, and the establishment of novel, easy-to-use, highly efficient, and accurate cloning methods remain a necessity.

## Author Contributions

WW and GZ wrote the draft. YL edited the manuscript. All the authors contributed to the article and approved the submitted version.

## Conflict of Interest

The authors declare that the research was conducted in the absence of any commercial or financial relationships that could be construed as a potential conflict of interest.

## References

[B1] Abbasi M. N. Fu J. Bian X. Wang H. Zhang Y. Li A. (2020). Recombineering for genetic engineering of natural product biosynthetic pathways. *Trends Biotechnol.* 38 715–728. 10.1016/j.tibtech.2019.12.018 31973879

[B2] Alberti F. Leng D. J. Wilkening I. Song L. Tosin M. Corre C. (2019). Triggering the expression of a silent gene cluster from genetically intractable bacteria results in scleric acid discovery. *Chem. Sci.* 10 453–463. 10.1039/c8sc03814g 30746093 PMC6335953

[B3] Aslanidis C. de Jong P. J. (1990). Ligation-independent cloning of PCR products (LIC-PCR). *Nucleic Acids Res.* 18 6069–6074. 10.1093/nar/18.20.6069 2235490 PMC332407

[B4] Atanasov A. G. Zotchev S. B. Dirsch V. M. Supuran T. C. (2021). Natural products in drug discovery: advances and opportunities. *Nat. Rev. Drug. Discov.* 20 200–216. 10.1038/s41573-020-00114-z 33510482 PMC7841765

[B5] Aubry C. Pernodet J. L. Lautru S. (2019). Modular and integrative vectors for synthetic biology applications in *Streptomyces* spp. *Appl. Environ. Microbiol.* 85:e00485-19. 10.1128/AEM.00485-19 31175189 PMC6677859

[B6] Bajpai B. (2014). *High Capacity Vectors.* Berlin: Springer.

[B7] Basitta P. Westrich L. Rösch M. Kulik A. Gust B. Apel A. K. (2017). AGOS: a plug-and-play method for the assembly of artificial gene operons into functional biosynthetic gene clusters. *ACS Chem. Biol.* 6 817–825. 10.1021/acssynbio.6b00319 28182401

[B8] Bayot Custodio A. Alcantara E. P. (2019). Identification of polyketide synthase gene clusters in a phage P1-derived artificial chromosome library of a Philippine strain of *Streptomyces* sp. PCS3-D2. *Asia Pac. J. Mol. Biol. Biotechnol.* 27 56–63. 10.35118/apjmbb.2019.027.2.08

[B9] Bennett-Baker P. E. Mueller J. L. (2017). CRISPR-mediated isolation of specific megabase segments of genomic DNA. *Nucleic Acids Res.* 45:e165. 10.1093/nar/gkx749 28977642 PMC5737698

[B10] Bilyk O. Sekurova O. N. Zotchev S. B. Luzhetskyy A. (2016). Cloning and heterologous expression of the grecocycline biosynthetic gene cluster. *PLoS One* 11:e0158682. 10.1371/journal.pone.0158682 27410036 PMC4943663

[B11] Binz T. M. Wenzel S. C. Schnell H. J. Bechthold A. Muller R. (2008). Heterologous expression and genetic engineering of the phenalinolactone biosynthetic gene cluster by using red/ET recombineering. *Chembiochem* 9 447–454. 10.1002/cbic.200700549 18157854

[B12] Bok J. W. Ye R. Clevenger K. D. Mead D. Wagner M. Krerowicz A. (2015). Fungal artificial chromosomes for mining of the fungal secondary metabolome. *BMC Genom.* 16:343. 10.1186/s12864-015-1561-x 25925221 PMC4413528

[B13] Bonet B. Teufel R. Crusemann M. Ziemert N. Moore B. S. (2015). Direct capture and heterologous expression of *Salinispora* natural product genes for the biosynthesis of enterocin. *J. Nat. Prod.* 78 539–542. 10.1021/np500664q 25382643 PMC4380194

[B14] Bourgouin C. Delecluse A. de la Torre F. Szulmajster J. (1990). Transfer of the toxin protein genes of *Bacillus sphaericus* into *Bacillus thuringiensis* subsp. israelensis and their expression. *Appl. Environ. Microbiol.* 56 340–344. 10.1128/AEM.56.2.340-344.1990 2306087 PMC183341

[B15] Burke D. T. Carle G. F. Olson M. V. (1987). Cloning of large segments of exogenous DNA into yeast by means of artificial chromosome vectors. *Science* 236 806–812. 10.1126/science.3033825 3033825

[B16] Casini A. MacDonald J. T. De Jonghe J. Christodoulou G. Freemont P. S. Baldwin G. S. (2014). One-pot DNA construction for synthetic biology: the modular Overlap-Directed Assembly with Linkers (MODAL) strategy. *Nucleic Acids Res.* 42:e7. 10.1093/nar/gkt915 24153110 PMC3874208

[B17] Chen W. H. Qin Z. J. Wang J. Zhao G. P. (2013). The MASTER (methylation-assisted tailorable ends rational) ligation method for seamless DNA assembly. *Nucleic Acids Res.* 41:e93. 10.1093/nar/gkt122 23444142 PMC3632120

[B18] Choi S. Nah H. J. Choi S. Kim E. S. (2019). Heterologous expression of daptomycin biosynthetic gene cluster via *Streptomyces* artificial chromosome vector system. *J. Microbiol. Biotechnol.* 29 1931–1937. 10.4014/jmb.1909.09022 31693835

[B19] Clevenger K. D. Bok J. W. Ye R. Miley G. P. Verdan M. H. Velk T. (2017). A scalable platform to identify fungal secondary metabolites and their gene clusters. *Nat. Chem. Biol.* 13 895–901. 10.1038/nchembio.2408 28604695 PMC5577364

[B20] Clos J. Zander-Dinse D. (2019). Cosmid library construction and functional cloning. *Methods Mol. Biol.* 1971 123–140. 10.1007/978-1-4939-9210-2_6 30980301

[B21] D’Agostino P. M. Gulder T. A. M. (2018). Direct pathway cloning combined with sequence- and ligation-independent cloning for fast biosynthetic gene cluster refactoring and heterologous expression. *ACS Synth. Biol.* 7 1702–1708. 10.1021/acssynbio.8b00151 29940102

[B22] Daniel R. (2005). The metagenomics of soil. *Nat. Rev. Microbiol.* 3 470–478. 10.1038/nrmicro1160 15931165

[B23] de Kok S. Stanton L. H. Slaby T. Durot M. Holmes V. F. Patel K. G. (2014). Rapid and reliable DNA assembly via ligase cycling reaction. *ACS Chem. Biol.* 3 97–106. 10.1021/sb4001992 24932563

[B24] Du D. Wang L. Tian Y. Liu H. Tan H. Niu G. (2015). Genome engineering and direct cloning of antibiotic gene clusters via phage φbT1 integrase-mediated site-specific recombination in *Streptomyces*. *Sci. Rep.* 5:8740. 10.1038/srep08740 25737113 PMC4349145

[B25] Enghiad B. Huang C. Guo F. Jiang G. Wang B. Tabatabaei S. K. (2021). Cas12a-assisted precise targeted cloning using *in vivo* Cre-lox recombination. *Nat. Commun.* 12:1171. 10.1038/s41467-021-21275-4 33608525 PMC7896053

[B26] Engler C. Gruetzner R. Kandzia R. Marillonnet S. (2009). Golden gate shuffling: a one-pot DNA shuffling method based on type IIs restriction enzymes. *PLoS One* 4:e5553. 10.1371/journal.pone.0005553 19436741 PMC2677662

[B27] Fogg P. C. Colloms S. Rosser S. Stark M. Smith M. C. (2014). New applications for phage integrases. *J. Mol. Biol.* 426 2703–2716. 10.1016/j.jmb.2014.05.014 24857859 PMC4111918

[B28] Fu J. Bian X. Hu S. Wang H. Huang F. Seibert P. M. (2012). Full-length RecE enhances linear-linear homologous recombination and facilitates direct cloning for bioprospecting. *Nat. Biotechnol.* 30 440–448. 10.1038/nbt.2183 22544021

[B29] Gao H. Taylor G. Evans S. K. Fogg P. C. M. Smith M. C. M. (2020). Application of serine integrases for secondary metabolite pathway assembly in *Streptomyces*. *Synth. Syst. Biotechnol.* 5 111–119. 10.1016/j.synbio.2020.05.006 32596521 PMC7306541

[B30] Gibson D. G. Young L. Chuang R. Y. Venter J. C. Hutchison C. A. Smith H. O. (2009). Enzymatic assembly of DNA molecules up to several hundred kilobases. *Nat. Methods* 6 343–345. 10.1038/nmeth.1318 19363495

[B31] Gomez-Acata E. S. Centeno C. M. Falcon L. I. (2019). Methods for extracting ‘omes from microbialites. *J. Microbiol. Methods* 160 1–10. 10.1016/j.mimet.2019.02.014 30877015

[B32] Greunke C. Duell E. R. D’Agostino P. M. Glöckle A. Lamm K. Gulder T. A. M. (2018). Direct Pathway Cloning (DiPaC) to unlock natural product biosynthetic potential. *Metab. Eng.* 47 334–345. 10.1016/j.ymben.2018.03.010 29548983

[B33] Grindley N. D. Whiteson K. L. Rice P. A. (2006). Mechanisms of site-specific recombination. *Annu. Rev. Biochem.* 75 567–605. 10.1146/annurev.biochem.73.011303.073908 16756503

[B34] Hashimoto T. Hashimoto J. Kozone I. Amagai K. Kawahara T. Takahashi S. (2018). Biosynthesis of quinolidomicin, the largest known macrolide of terrestrial origin: identification and heterologous expression of a biosynthetic gene cluster over 200 kb. *Org. Lett.* 20 7996–7999. 10.1021/acs.orglett.8b03570 30543302

[B35] Hover B. M. Kim S. H. Katz M. Charlop-Powers Z. Owen J. G. Ternei M. A. (2018). Culture-independent discovery of the malacidins as calcium-dependent antibiotics with activity against multidrug-resistant Gram-positive pathogens. *Nat. Microbiol.* 3 415–422. 10.1038/s41564-018-0110-1 29434326 PMC5874163

[B36] Hu S. Liu Z. Zhang X. Zhang G. Xie Y. Ding X. (2016). “Cre/loxP plus BAC”: a strategy for direct cloning of large DNA fragment and its applications in *Photorhabdus luminescens* and *Agrobacterium tumefaciens*. *Sci. Rep.* 6:29087. 10.1038/srep29087 27364376 PMC4929569

[B37] Hu Y. Nan F. Maina S. W. Guo J. Wu S. Xin Z. (2018). Clone of plipastatin biosynthetic gene cluster by transformation-associated recombination technique and high efficient expression in model organism *Bacillus subtilis*. *J. Biotechnol.* 288 1–8. 10.1016/j.jbiotec.2018.10.006 30343036

[B38] Huang F. Tang J. He L. Ding X. Huang S. Zhang Y. (2018). Heterologous expression and antitumor activity analysis of syringolin from *Pseudomonas syringae* pv. syringae B728a. *Microb. Cell Fact.* 17:31. 10.1186/s12934-018-0859-1 29482589 PMC6389232

[B39] Ignatov K. B. Blagodatskikh K. A. Shcherbo D. S. Kramarova T. V. Monakhova Y. A. Kramarov V. M. (2019). Fragmentation through polymerization (FTP): a new method to fragment DNA for next-generation sequencing. *PLoS One* 14:e0210374. 10.1371/journal.pone.0210374 30933980 PMC6443234

[B40] Ioannou P. A. de P. J. Jong (1996). *Current Protocols in Human Genetics.* Hoboken, NJ: Wiley.

[B41] Jiang W. Zhao X. Gabrieli T. Lou C. Ebenstein Y. Zhu T. F. (2015). Cas9-Assisted targeting of CHromosome segments CATCH enables one-step targeted cloning of large gene clusters. *Nat. Commun.* 6:8101. 10.1038/ncomms9101 26323354 PMC4569707

[B42] Jiang W. Zhu T. F. (2016). Targeted isolation and cloning of 100-kb microbial genomic sequences by Cas9-assisted targeting of chromosome segments. *Nat. Protoc.* 11 960–975. 10.1038/nprot.2016.055 27101517

[B43] Jin P. Ding W. Du G. Chen J. Kang Z. (2016). DATEL: a scarless and sequence-independent DNA assembly method using thermostable exonucleases and ligase. *ACS Chem. Biol.* 5 1028–1032. 10.1021/acssynbio.6b00078 27230689

[B44] Jones A. C. Gust B. Kulik A. Heide L. Buttner M. J. Bibb M. J. (2013). Phage P1-derived artificial chromosomes facilitate heterologous expression of the FK506 gene cluster. *PLoS One* 8:e69319. 10.1371/journal.pone.0069319 23874942 PMC3708917

[B45] Jordan P. A. Moore B. S. (2016). Biosynthetic pathway connects cryptic ribosomally synthesized posttranslationally modified peptide genes with pyrroloquinoline alkaloids. *Cell Chem. Biol.* 23 1504–1514. 10.1016/j.chembiol.2016.10.009 27866908 PMC5182094

[B46] Juhas M. Ajioka J. W. (2017). High molecular weight DNA assembly *in vivo* for synthetic biology applications. *Crit. Rev. Biotechnol.* 37 277–286. 10.3109/07388551.2016.1141394 26863154

[B47] Kang H. S. Kim E. S. (2021). Recent advances in heterologous expression of natural product biosynthetic gene clusters in *Streptomyces hosts*. *Curr. Opin. Biotechnol.* 69 118–127. 10.1016/j.copbio.2020.12.016 33445072

[B48] Kim J. H. Feng Z. Bauer J. D. Kallifidas D. Calle P. Y. Brady S. F. (2010). Cloning large natural product gene clusters from the environment: piecing environmental DNA gene clusters back together with TAR. *Biopolymers* 93 833–844. 10.1002/bip.21450 20577994 PMC2895911

[B49] Kim U. J. Shizuya H. de Jong P. J. Birren B. Simon M. I. (1992). Stable propagation of cosmid sized human DNA inserts in an F factor based vector. *Nucleic Acids Res.* 20 1083–1085. 10.1093/nar/20.5.1083 1549470 PMC312094

[B50] Kouprina N. Larionov V. (2016). Transformation-associated recombination (TAR) cloning for genomics studies and synthetic biology. *Chromosoma* 125 621–632. 10.1007/s00412-016-0588-3 27116033 PMC5025352

[B51] Kouprina N. Larionov V. (2019). TAR cloning: perspectives for functional genomics, biomedicine, and biotechnology. *Mol. Ther. Methods Clin. Dev.* 14 16–26. 10.1016/j.omtm.2019.05.006 31276008 PMC6586605

[B52] Kouprina N. Noskov V. N. Larionov V. (2006). Selective isolation of large chromosomal regions by transformation-associated recombination cloning for structural and functional analysis of mammalian genomes. *Methods Mol. Biol.* 349 85–101. 10.1385/1-59745-158-4 17071976

[B53] Kouprina N. Noskov V. N. Larionov V. (2020). Selective isolation of large segments from individual microbial genomes and environmental DNA samples using transformation-associated recombination cloning in yeast. *Nat. Protocols* 15 734–749. 10.1038/s41596-019-0280-1 32005981 PMC7440688

[B54] Kunes S. Botstein D. Fox M. S. (1985). Transformation of yeast with linearized plasmid DNA: formation of inverted dimers and recombinant plasmid products. *J. Mol. Biol.* 184 375–387. 10.1016/0022-2836(85)90288-8 3900413

[B55] Larionov V. Kouprina N. Solomon G. Barrett J. C. Resnick M. A. (1997). Direct isolation of human brca2 gene by transformation-associated recombination in yeast. *Proc. Natl. Acad. Sci. U S A.* 94 7384–7387. 10.1073/pnas.94.14.7384 9207100 PMC23830

[B56] Larson C. B. Crusemann M. Moore B. S. (2017). PCR-independent method of transformation-associated recombination reveals the cosmomycin biosynthetic gene cluster in an ocean *Streptomycete*. *J. Nat. Prod.* 80 1200–1204. 10.1021/acs.jnatprod.6b01121 28333450 PMC5714584

[B57] Lee N. C. O. Larionov V. Kouprina N. (2015). Highly efficient CRISPR/Cas9-mediated TAR cloning of genes and chromosomal loci from complex genomes in yeast. *Nucleic Acids Res.* 43:e55. 10.1093/nar/gkv112 25690893 PMC4417148

[B58] Lei C. Li S. Y. Liu J. K. Zheng X. Zhao G. P. Wang J. (2017). The CCTL (Cpf1-assisted cutting and Taq DNA ligase-assisted Ligation) method for efficient editing of large DNA constructs *in vitro*. *Nucleic Acids Res.* 45:e74. 10.1093/nar/gkx018 28115632 PMC5436000

[B59] Lesic B. Rahme L. G. (2008). Use of the lambda Red recombinase system to rapidly generate mutants in *Pseudomonas aeruginosa*. *BMC Mol. Biol.* 9:20. 10.1186/1471-2199-9-20 18248677 PMC2287187

[B60] Lewis W. H. Tahon G. Geesink P. Sousa D. Z. Ettema T. J. G. (2021). Innovations to culturing the uncultured microbial majority. *Nat. Rev. Microbiol.* 19 225–240. 10.1038/s41579-020-00458-8 33093661

[B61] Li L. Jiang W. H. Lu Y. H. (2017). New strategies and approaches for engineering biosynthetic gene clusters of microbial natural products. *Biotechnol. Adv.* 35 936–949. 10.1016/j.biotechadv.2017.03.007 28323062

[B62] Li L. Maclntyre L. W. Brady S. F. (2021). Refactoring biosynthetic gene clusters for heterologous production of microbial natural products. *Curr. Opin. Biotechnol.* 69 145–152. 10.1016/j.copbio.2020.12.011 33476936 PMC8238852

[B63] Li L. Zhao Y. Ruan L. Yang S. Ge M. Jiang W. (2015). A stepwise increase in pristinamycin II biosynthesis by *Streptomyces pristinaespiralis* through combinatorial metabolic engineering. *Metab. Eng.* 29 12–25. 10.1016/j.ymben.2015.02.001 25708513

[B64] Li M. Z. Elledge S. J. (2007). Harnessing homologous recombination *in vitro* to generate recombinant DNA via SLIC. *Nat. Methods* 4 251–256. 10.1038/nmeth1010 17293868

[B65] Li M. Z. Elledge S. J. (2012). SLIC: a method for sequence- and ligation-independent cloning. *Methods Mol. Biol.* 852 51–59. 10.1007/978-1-61779-564-0_5 22328425

[B66] Li Y. Li Z. Yamanaka K. Xu Y. Zhang W. Vlamakis H. (2015). Directed natural product biosynthesis gene cluster capture and expression in the model bacterium *Bacillus subtilis*. *Sci. Rep.* 5:9383. 10.1038/srep09383 25807046 PMC4894447

[B67] Liang J. Liu Z. Low X. Z. Ang E. L. Zhao H. (2017). Twin-primer non-enzymatic DNA assembly: an efficient and accurate multi-part DNA assembly method. *Nucleic Acids Res.* 45:e94. 10.1093/nar/gkx132 28334760 PMC5499748

[B68] Libis V. Antonovsky N. Zhang M. Shang Z. Montiel D. Maniko J. (2019). Uncovering the biosynthetic potential of rare metagenomic DNA using co-occurrence network analysis of targeted sequences. *Nat. Commun.* 10:3848. 10.1038/s41467-019-11658-z 31451725 PMC6710260

[B69] Lin Z. Nielsen J. Liu Z. (2020). Bioprospecting through cloning of whole natural product biosynthetic gene clusters. *Front. Bioeng. Biotechnol.* 8:526. 10.3389/fbioe.2020.00526 32582659 PMC7290108

[B70] Liu C. J. Jiang H. Wu L. Zhu L. Y. Meng E. Zhang D. Y. (2017). OEPR cloning: an efficient and seamless cloning strategy for large- and multi-fragments. *Sci. Rep.* 7:44648. 10.1038/srep44648 28300166 PMC5353728

[B71] Liu H. Jiang H. Haltli B. Kulowski K. Muszynska E. Feng X. (2009). Rapid cloning and heterologous expression of the meridamycin biosynthetic gene cluster using a versatile *Escherichia coli*-Streptomyces artificial chromosome vector, pSBAC. *J. Nat. Prod.* 72 389–395. 10.1021/np8006149 19191550

[B72] Liu Q. Shen Q. Bian X. Chen H. Fu J. Wang H. (2016). Simple and rapid direct cloning and heterologous expression of natural product biosynthetic gene cluster in *Bacillus subtilis* via Red/ET recombineering. *Sci. Rep.* 6:34623. 10.1038/srep34623 27687863 PMC5043344

[B73] Liu T. Mazmouz R. Ongley S. E. Chau R. Pickford R. Woodhouse J. N. (2017). Directing the heterologous production of specific cyanobacterial toxin variants. *ACS Chem. Biol.* 12 2021–2029. 10.1021/acschembio.7b00181 28570054

[B74] Loannou P. A. Amemiya C. T. Garnes J. Kroisel P. M. Shizuya H. Chen C. (1994). A new bacteriophage P1–derived vector for the propagation of large human DNA fragments. *Nat. Genet.* 6 84–89. 10.1038/ng0194-84 8136839

[B75] Ma H. Kunes S. Schatz P. J. Botstein D. (1987). Plasmid construction by homologous recombination in yeast. *Gene* 58 201–216. 10.1016/0378-1119(87)90376-3 2828185

[B76] Marillonnet S. Grutzner R. (2020). Synthetic DNA assembly using golden gate cloning and the hierarchical modular cloning pipeline. *Curr. Protoc. Mol. Biol.* 130:e115. 10.1002/cpmb.115 32159931

[B77] Martinez A. Kolvek S. J. Yip C. L. T. Hopke J. Brown K. A. (2004). Genetically modified bacterial strains and novel bacterial artificial chromosome shuttle vectors for constructing environmental libraries and detecting heterologous natural products in multiple expression hosts. *Appl. Environ. Microbiol.* 70 2452–2463. 10.1128/AEM.70.4.2452-2463.2004 15066844 PMC383137

[B78] Martinez-Garcia E. Goni-Moreno A. Bartley B. McLaughlin J. Sanchez-Sampedro L. Pascual Del H. (2020). SEVA 3.0: an update of the standard european vector architecture for enabling portability of genetic constructs among diverse bacterial hosts. *Nucleic Acids Res.* 48 D1164–D1170. 10.1093/nar/gkz1024 31740968 PMC7018797

[B79] Mayjonade B. Gouzy J. Donnadieu C. Pouilly N. Marande W. Callot C. (2016). Extraction of high-molecular-weight genomic DNA for long-read sequencing of single molecules. *Biotechniques* 61 203–205. 10.2144/000114460 27712583

[B80] Meng J. Feng R. Zheng G. Ge M. Mast Y. Wohlleben W. (2017). Improvement of pristinamycin I (PI) production in *Streptomyces pristinaespiralis* by metabolic engineering approaches. *Synth. Syst. Biotechnol.* 2 130–136. 10.1016/j.synbio.2017.06.001 29062970 PMC5636943

[B81] Miao V. Coeffet-LeGal M. F. Brian P. Brost R. Penn J. Whiting A. (2005). Daptomycin biosynthesis in *Streptomyces roseosporus*: cloning and analysis of the gene cluster and revision of peptide stereochemistry. *Microbiology* 151 1507–1523. 10.1099/mic.0.27757-0 15870461

[B82] Milon N. Chantry-Darmon C. Satge C. Fustier M. A. Cauet S. Moreau S. (2019). muLAS technology for DNA isolation coupled to Cas9-assisted targeting for sequencing and assembly of a 30 kb region in plant genome. *Nucleic Acids Res.* 47 8050–8060. 10.1093/nar/gkz632 31505675 PMC6736094

[B83] Mitchell L. A. Chuang J. Agmon N. Khunsriraksakul C. Phillips N. A. Cai Y. (2015). Versatile genetic assembly system (VEGAS) to assemble pathways for expression in *S. cerevisiae*. *Nucleic Acids Res.* 43 6620–6630. 10.1093/nar/gkv466 25956652 PMC4513848

[B84] Monaco A. P. Larin Z. (1994). YACs, BACs, PACs and MACs: artificial chromosomes as research tools. *Trends Biotechnol.* 12 280–286. 10.1016/0167-7799(94)90140-6 7765076

[B85] Myronovskyi M. Luzhetskyy A. (2013). Genome engineering in actinomycetes using site-specific recombinases. *Appl. Microbiol. Biotechnol.* 97 4701–4712. 10.1007/s00253-013-4866-1 23584280

[B86] Nah H. J. Woo M. W. Choi S. S. Kim E. S. (2015). Precise cloning and tandem integration of large polyketide biosynthetic gene cluster using *Streptomyces* artificial chromosome system. *Microb. Cell Fact.* 14:140. 10.1186/s12934-015-0325-2 26377404 PMC4573296

[B87] Noskov V. Kouprina N. Leem S. H. Koriabine M. Barrett J. C. Larionov V. (2002). A genetic system for direct selection of gene-positive clones during recombinational cloning in yeast. *Nucleic Acids Res.* 30:E8. 10.1093/nar/30.2.e8 11788734 PMC99847

[B88] Noskov V. N. Chuang R. Y. Gibson D. G. Leem S. H. Larionov V. Kouprina N. (2011). Isolation of circular yeast artificial chromosomes for synthetic biology and functional genomics studies. *Nat. Protoc.* 6 89–96. 10.1038/nprot.2010.174 21212778 PMC7380547

[B89] Noskov V. N. Kouprina N. Leem S. H. Ouspenski I. Barrett C. Larionov V. (2003). A general cloning system to selectively isolate any eukaryotic or prokaryotic genomic region in yeast. *BMC Genomics* 4:16. 10.1186/1471-2164-4-16 12720573 PMC156606

[B90] Oliner J. D. Kinzler K. W. Vogelstein B. (1993). *In vivo* cloning of PCR products in *E. coli*. *Nucleic Acids Res.* 21 5192–5197. 10.1093/nar/21.22.5192 8255776 PMC310636

[B91] Park D. (2007). Genomic DNA isolation from different biological materials. *Methods Mol. Biol.* 353 3–13. 10.1385/1-59745-229-7:3 17332629

[B92] Pedersen T. B. Nielsen M. R. Kristensen S. B. Spedtsberg E. M. L. Yasmine W. Matthiesen R. (2020). Heterologous expression of the core genes in the complex fusarubin gene cluster of Fusarium Solani. *Int. J. Mol. Sci.* 21:7601. 10.3390/ijms21207601 33066643 PMC7589453

[B93] Penouilh-Suzette C. Fourre S. Besnard G. Godiard L. Pecrix Y. (2020). A simple method for high molecular-weight genomic DNA extraction suitable for long-read sequencing from spores of an obligate biotroph oomycete. *J. Microbiol. Methods* 178:106054. 10.1016/j.mimet.2020.106054 32926900

[B94] Phelan R. M. Sachs D. Petkiewicz S. J. Barajas J. F. Blake-Hedges J. M. Thompson M. G. (2017). Development of next generation synthetic biology tools for use in *Streptomyces venezuelae*. *ACS Chem. Biol.* 6 159–166. 10.1021/acssynbio.6b00202 27605473

[B95] Pyeon H. R. Nah H. J. Kang S. H. Choi S. S. Kim E. S. (2017). Heterologous expression of pikromycin biosynthetic gene cluster using *Streptomyces artificial* chromosome system. *Microb. Cell Fact.* 16:96. 10.1186/s12934-017-0708-7 28569150 PMC5452415

[B96] Qian Z. Bruhn T. D’Agostino P. M. Herrmann A. Haslbeck M. Antal N. (2020). Discovery of the Streptoketides by direct cloning and rapid heterologous expression of a cryptic PKS II gene cluster from *Streptomyces* sp. Tü 6314. *J. Org. Chem.* 85 664–673. 10.1021/acs.joc.9b02741 31746205

[B97] Quan J. Tian J. (2014). Circular p polymerase extension cloning. *Methods Mol. Biol.* 1116 103–117. 10.1007/978-1-62703-764-8_8 24395360

[B98] Ramsay M. (1994). Yeast artificial chromosome cloning. *Mol. Biotechnol.* 1 181–201. 10.1007/BF02921558 7859160

[B99] Ray L. Yamanaka K. Moore B. S. (2016). A peptidyl-transesterifying type I thioesterase in salinamide biosynthesis. *Angew. Chem. Int. Ed. Engl.* 55 364–367. 10.1002/anie.201508576 26553755 PMC4715598

[B100] Ren H. Hu P. Zhao H. (2017). A plug-and-play pathway refactoring workflow for natural product research in *Escherichia coli* and *Saccharomyces cerevisiae*. *Biotechnol. Bioeng.* 114 1847–1854. 10.1002/bit.26309 28401530 PMC5500230

[B101] Richter D. Bayer K. Toesko T. Schuster S. (2019). ZeBRalpha a universal, multi-fragment DNA-assembly-system with minimal hands-on time requirement. *Sci. Rep.* 9:2980. 10.1038/s41598-019-39768-0 30814590 PMC6393441

[B102] Ross A. C. Gulland L. E. S. Dorrestein P. C. Moore B. S. (2015). Targeted capture and heterologous expression of the pseudoalteromonas alterochromide gene cluster in *Escherichia coil* represents a promising natural product exploratory platform. *ACS Chem. Biol.* 4 414–420. 10.1021/sb500280q 25140825 PMC4410906

[B103] Salomonsen B. Mortensen U. H. Halkier B. A. (2014). USER-derived cloning methods and their primer design. *Methods Mol. Biol.* 1116 59–72. 10.1007/978-1-62703-764-8_5 24395357

[B104] Sapojnikova N. Asatiani N. Kartvelishvili T. Asanishvili L. Zinkevich V. Bogdarina I. (2017). A comparison of DNA fragmentation methods - applications for the biochip technology. *J. Biotechnol.* 256 1–5. 10.1016/j.jbiotec.2017.06.1202 28666852

[B105] Schlichting N. Reinhardt F. Jager S. Schmidt M. Kabisch J. (2019). Optimization of the experimental parameters of the ligase cycling reaction. *Synth. Biol.* 4:ysz020. 10.1093/synbio/ysz020 32995543 PMC7445781

[B106] Schmid-Burgk J. L. Schmidt T. Kaiser V. Honing K. Hornung V. (2013). A ligation-independent cloning technique for high-throughput assembly of transcription activator-like effector genes. *Nat. Biotechnol.* 31 76–81. 10.1038/nbt.2460 23242165 PMC4142318

[B107] Scott H. N. Laible P. D. Hanson D. K. (2003). Sequences of versatile broad-host-range vectors of the RK2 family. *Plasmid* 50 74–79. 10.1016/s0147-619x(03)00030-1 12826060

[B108] Shizuya H. Birren B. Kim U. J. Mancino V. Slepak T. Tachiiri Y. (1992). Cloning and stable maintenance of 300-kilobase-pair fragments of human DNA in *Escherichia coli* using an F-factor-based vector. *Proc. Natl. Acad. Sci. U S A.* 89 8794–8797. 10.13/pnas.89.18.8794 1528894 PMC50007

[B109] Shizuya H. Kouros-Mehr H. (2001). The development and applications of the bacterial artificial chromosome cloning system. *Keio J. Med.* 50 26–30. 10.2302/kjm.50.26 11296661

[B110] Song C. Luan J. Cui Q. Duan Q. Li Z. Gao Y. (2019). Enhanced heterologous spinosad production from a 79-kb synthetic multioperon assembly. *ACS Chem. Biol.* 8 137–147. 10.1021/acssynbio.8b00402 30590919

[B111] Sosio M. Giusino F. Cappellano C. Bossi E. Puglia A. M. Donadio S. (2000). Artificial chromosomes for antibiotic-producing actinomycetes. *Nat. Biotechnol.* 18 343–345. 10.1038/73810 10700154

[B112] Stinchcomb D. T. Thomas M. Kelly J. Selker E. Davis R. W. (1980). Eukaryotic DNA segments capable of autonomous replication in yeast. *Proc. Natl. Acad. Sci. U S A.* 77 4559–4563. 10.1073/pnas.77.8.4559 6449009 PMC349883

[B113] Sun F. Xu S. Jiang F. Liu W. (2018). Genomic-driven discovery of an amidinohydrolase involved in the biosynthesis of mediomycin A. *Appl. Microbiol. Biotechnol.* 102 2225–2234. 10.1007/s00253-017-8729-z 29349495

[B114] Tang X. Li J. Millán-Aguiñaga N. Zhang J. J. O’Neill E. C. Ugalde J. A. (2015). Identification of thiotetronic acid antibiotic biosynthetic pathways by target-directed genome mining. *ACS Chem. Biol.* 10 2841–2849. 10.1021/acschembio.5b00658 26458099 PMC4758359

[B115] Tang Y. Frewert S. Harmrolfs K. Herrmann J. Karmann L. Kazmaier U. (2015). Heterologous expression of an orphan NRPS gene cluster from *Paenibacillus larvae* in *Escherichia coli* revealed production of sevadicin. *J. Biotechnol.* 194 112–114. 10.1016/j.jbiotec.2014.12.008 25529345

[B116] Tillett D. Neilan B. A. (1999). Enzyme-free cloning: a rapid method to clone PCR products independent of vector restriction enzyme sites. *Nucleic Acids Res.* 27:e26. 10.1093/nar/27.19.e26 10481038 PMC148636

[B117] Tu Q. Herrmann J. Hu S. Raju R. Bian X. Zhang Y. (2016). Genetic engineering and heterologous expression of the disorazol biosynthetic gene cluster via Red/ET recombineering. *Sci. Rep.* 6:21066. 10.1038/srep21066 26875499 PMC4753468

[B118] Varma A. Padh H. Shrivastava N. (2007). Plant genomic DNA isolation: an art or a science. *Biotechnol. J.* 2 386–392. 10.1002/biot.200600195 17285676

[B119] Wang H. Li Z. Jia R. Hou Y. Yin J. Bian X. (2016). RecET direct cloning and red alpha beta recombineering of biosynthetic gene clusters, large operons or single genes for heterologous expression. *Nat. Protoc.* 11 1175–1190. 10.1038/nprot.2016.054 27254463

[B120] Wang H. Li Z. Jia R. Yin J. Li A. Xia L. (2018). ExoCET: exonuclease *in vitro* assembly combined with RecET recombination for highly efficient direct DNA cloning from complex genomes. *Nucleic Acids Res.* 46:e28. 10.1093/nar/gkx1249 29240926 PMC5861427

[B121] Wang L. Wang H. Liu H. Zhao Q. Liu B. Wang L. (2019). Improved CRISPR-Cas12a-assisted one-pot DNA editing method enables seamless DNA editing. *Biotechnol. Bioeng.* 116, 1463–1474. 10.1002/bit.26938 30730047

[B122] Wenzel S. C. Gross F. Zhang Y. Fu J. Stewart A. F. Muller R. (2005). Heterologous expression of a myxobacterial natural products assembly line in pseudomonads via red/ET recombineering. *Chem. Biol.* 12 349–356. 10.1016/j.chembiol.2004.12.012 15797219

[B123] Wu C. Shang Z. Lemetre C. Ternei M. A. Brady S. F. (2019). Cadasides, calcium-dependent acidic lipopeptides from the soil metagenome that are active against multidrug-resistant bacteria. *J. Am. Chem. Soc.* 141 3910–3919. 10.1021/jacs.8b12087 30735616 PMC6592427

[B124] Wu N. Huang H. Min T. Hu H. (2017). TAR cloning and integrated overexpression of 6-demethylchlortetracycline biosynthetic gene cluster in *Streptomyces aureofaciens*. *Acta Biochim. Biophys. Sin.* 49 1129–1134. 10.1093/abbs/gmx110 29087452

[B125] Xia Y. Li K. Li J. Wang T. Gu L. Xun L. (2019). T5 exonuclease-dependent assembly offers a low-cost method for efficient cloning and site-directed mutagenesis. *Nucleic Acids Res.* 47:e15. 10.1093/nar/gky1169 30462336 PMC6379645

[B126] Xu P. Modavi C. Demaree B. Twigg F. Liang B. Sun C. (2020). Microfluidic automated plasmid library enrichment for biosynthetic gene cluster discovery. *Nucleic Acids Res.* 48:e48. 10.1093/nar/gkaa131 32095820 PMC7192590

[B127] Xu X. Zhou H. Liu Y. Liu X. Fu J. Li A. (2018). Heterologous expression guides identification of the biosynthetic gene cluster of chuangxinmycin, an indole alkaloid antibiotic. *J. Nat. Prod.* 81 1060–1064. 10.1021/acs.jnatprod.7b00835 29565122

[B128] Yamanaka K. Reynolds K. A. Kersten R. D. Ryan K. S. Gonzalez D. J. Nizet V. (2014). Direct cloning and refactoring of a silent lipopeptide biosynthetic gene cluster yields the antibiotic taromycin A. *Proc. Natl. Acad. Sci. U S A.* 111 1957–1962. 10.1073/pnas.1319584111 24449899 PMC3918841

[B129] Yamanaka K. Ryan K. S. Gulder T. A. Hughes C. C. Moore B. S. (2012). Flavoenzyme-catalyzed atropo-selective N,C-bipyrrole homocoupling in marinopyrrole biosynthesis. *J. Am. Chem. Soc.* 134 12434–12437. 10.1021/ja305670f 22800473 PMC3415713

[B130] Yin J. Hoffmann M. Bian X. Tu Q. Yan F. Xia L. (2015). Direct cloning and heterologous expression of the salinomycin biosynthetic gene cluster from Streptomyces albus DSM41398 in *Streptomyces coelicolor* A3(2). *Sci. Rep.* 5:15081. 10.1038/srep15081 26459865 PMC4602208

[B131] Yuan Y. Andersen E. Zhao H. (2016). Flexible and versatile strategy for the construction of large biochemical pathways. *ACS Chem. Biol.* 5 46–52. 10.1021/acssynbio.5b00117 26332374

[B132] Zeng F. Zang J. Zhang S. Hao Z. Dong J. Lin Y. (2017). AFEAP cloning: a precise and efficient method for large DNA sequence assembly. *BMC Biotechnol.* 17:81. 10.1186/s12896-017-0394-x 29137618 PMC5686892

[B133] Zhang J. J. Tang X. Zhang M. Nguyen D. Moore B. S. (2017). Broad-host-range expression reveals native and host regulatory elements that influence heterologous antibiotic production in Gram-negative bacteria. *mBio* 8:e01291-17. 10.1128/mBio.01291-17 28874475 PMC5587914

[B134] Zhang L. Hashimoto T. Qin B. Hashimoto J. Kozone I. Kawahara T. (2017). Characterization of giant modular PKSs provides insight into genetic mechanism for structural diversification of aminopolyol polyketides. *Angew Chem. Int. Ed. Engl.* 56 1740–1745. 10.1002/anie.201611371 28133950

[B135] Zhang M. Zhang Y. Scheuring C. F. Wu C. C. Dong J. J. Zhang H. B. (2012). Preparation of megabase-sized DNA from a variety of organisms using the nuclei method for advanced genomics research. *Nat. Protoc.* 7 467–478. 10.1038/nprot.2011.455 22343429

[B136] Zhang Y. Buchholz F. Muyrers J. P. Stewart A. F. (1998). A new logic for DNA engineering using recombination in *Escherichia coli*. *Nat. Genet.* 20 123–128. 10.1038/2417 9771703

[B137] Zhang Y. Werling U. Edelmann W. (2012). SLiCE: a novel bacterial cell extract-based DNA cloning method. *Nucleic Acids Res.* 40:e55. 10.1093/nar/gkr1288 22241772 PMC3333860

[B138] Zheng J. Li Y. Guan H. Zhang J. Tan H. (2019). Enhancement of neomycin production by engineering the entire biosynthetic gene cluster and feeding key precursors in *Streptomyces fradiae* CGMCC 4.576. *Appl. Microbiol. Biotechnol.* 103 2263–2275. 10.1007/s00253-018-09597-8 30685809

[B139] Zheng X. Cheng Q. Yao F. Wang X. Kong L. Cao B. (2017). Biosynthesis of the pyrrolidine protein synthesis inhibitor anisomycin involves novel gene ensemble and cryptic biosynthetic steps. *Proc. Natl. Acad. Sci. U S A.* 114 4135–4140. 10.1073/pnas.1701361114 28373542 PMC5402462

[B140] Ziko L. Saqr A. A. Ouf A. Gimpel M. Aziz R. K. Neubauer P. (2019). Antibacterial and anticancer activities of orphan biosynthetic gene clusters from atlantis II red sea brine pool. *Microb. Cell Fact.* 18:56. 10.1186/s12934-019-1103-3 30885206 PMC6423787

